# *SGK1* Is Upregulated in Retained Placenta and Mediates Estradiol Effects in Bovine Endometrial Cells

**DOI:** 10.3390/cells15060558

**Published:** 2026-03-20

**Authors:** Ruiqing Wang, Meng Wei, Wei Niu, Jingxiao Chen, Jinghong Nan, Yong Zhang, Xingxu Zhao, Qi Wang

**Affiliations:** 1 College of Veterinary Medicine, Gansu Agricultural University, Lanzhou 730070, China; 17352255912@163.com (R.W.); zhangyong@gsau.edu.cn (Y.Z.); 2 Gansu Key Laboratory of Animal Generational Physiology and Reproductive Regulation, Lanzhou 730070, China

**Keywords:** retained placenta, estradiol, serum and glucocorticoid-regulated kinase 1, dairy cows, apoptosis, tight junctions, cell migration

## Abstract

**Highlights:**

**What are the main findings?**

- *SGK1* is upregulated in retained placenta of dairy cows and correlates with suppressed apoptosis, increased tight junction proteins, and enhanced epithelial marker expression.
- Estradiol upregulates *SGK1* in bovine endometrial epithelial cells, and knockdown of this kinase abolishes estradiol effects on apoptosis, junctional protein expression, and cell migration.

**What are the implications of the main findings?**

- The findings propose a new mechanistic hypothesis: sustained estradiol–*SGK1* signaling may excessively stabilize the fetomaternal interface, contributing to retained placenta.
- *SGK1* is a candidate tissue biomarker for retained placenta, providing a foundation for future validation and translational studies.

**Abstract:**

Retained placenta (RP) is a significant postpartum complication in dairy cows. Although abnormal estradiol (E_2_) levels are implicated, the underlying cellular mechanisms remain poorly defined. Through RNA-seq analysis of postpartum blood from cows with or without RP, we identified Serum and Glucocorticoid-regulated Kinase 1 (*SGK1*) as a differentially expressed gene candidate. Analysis of fetal cotyledonary tissues revealed that *SGK1* expression was significantly elevated in these tissues, concomitant with markers of suppressed apoptosis, increased levels of tight junction proteins, and an inhibited epithelial–mesenchymal transition (EMT) phenotype. To explore a potential mechanistic link between E_2_ and these cellular alterations, we investigated the E_2_-*SGK1* axis in bovine endometrial epithelial cells in vitro. E_2_ treatment upregulated *SGK1* expression, reduced apoptosis, increased tight junction protein levels, and suppressed EMT. Conversely, *SGK1* knockdown induced apoptosis, disrupted tight junctions, and impaired EMT. Notably, E_2_ could not rescue the apoptosis and EMT alterations in *SGK1*-knockdown cells, indicating that *SGK1* is a critical mediator of these E_2_ effects in this cellular model. Based on these initial correlative findings in tissues, combined with the subsequent mechanistic experiments in cells, we propose a novel model whereby dysregulation of the E_2_- *SGK1* axis could contribute to RP pathogenesis by stabilizing the placental interface. Our findings provide the first experimental evidence linking *SGK1* to RP and establish a foundation for future in vivo validation.

## 1. Introduction

Retained placenta (RP), defined as the failure to expel fetal membranes within 12 h post-calving [1,2], is a major periparturient condition in dairy cows with substantial economic consequences, including reduced milk yield, secondary infections such as metritis, and impaired fertility [3,4,5]. The bovine placenta is of the cotyledonary type, formed by the interlocking of maternal endometrial caruncles and fetal cotyledons, where fetal villi embed within maternal glandular crypts [6,7,8]. In ruminants, the timely detachment of this interface is a programmed physiological event, dependent on the coordinated apoptosis of trophoblast and endometrial epithelial cells and the disassembly of their intercellular junctions [6,7,9], and is highly regulated by periparturient endocrine signals, including glucocorticoids, estradiol, progesterone, and metabolic hormones [10,11]. A disruption in this precisely coordinated sequence prevents normal villous detachment, leading to RP; however, the underlying molecular mechanisms remain incompletely understood.

Transcriptomic approaches, such as RNA sequencing (RNA-seq), have been instrumental in identifying molecular changes associated with RP [12,13]. Building on these findings, we employed RNA-seq to screen for novel candidate genes involved in the disorder.

Hormonal dysregulation, particularly of estradiol (E_2_), is strongly implicated in RP [14]. E_2_ surges before parturition and is a critical initiator of delivery [15]. It regulates diverse processes essential for placental release, including apoptosis, inflammation, and extracellular matrix remodeling [16,17]. Notably, abnormal periparturient circulatory E_2_ profiles are consistently observed in cows with RP [13,18], yet the key downstream effectors translating this hormonal signal into failed detachment are largely unknown.

Serum and Glucocorticoid-Regulated Kinase 1 (SGK1) is an AGC family kinase that integrates diverse extracellular signals, including hormonal cues [19,20,21]. In reproduction, *SGK1* is involved in endometrial receptivity, embryo implantation, and placental development in humans and rodents [22,23,24]. However, its expression, regulation, and function in ruminant reproduction, particularly during the periparturient period, remain completely unexplored. *SGK1* regulates fundamental cellular processes such as apoptosis, ion transport, cell survival, and fluid homeostasis [25,26,27]. While *SGK1* is a recognized effector of progesterone signaling [28], its potential role in estrogen signaling and in the terminal event of placental separation—particularly in dairy cows—has never been investigated.

Therefore, we hypothesized that *SGK1* might function as a novel molecular node linking aberrant E_2_ signaling to the cellular pathogenesis of RP. To bridge systemic signals and local tissue pathology, we employed an integrated strategy. First, we leveraged the postpartum blood RNA-seq dataset as a discovery platform to bioinformatically screen for candidate genes functionally linked to processes like hormone response and cell adhesion. *SGK1* was identified as a prime candidate through this screening. Through RNA-seq analysis of tail vein blood from postpartum cows, we identified *SGK1* as a differentially expressed candidate, followed by validation of its expression in RP fetal cotyledonary tissues. We then utilized a bovine endometrial epithelial cell model to interrogate whether E_2_ regulates *SGK1* and whether *SGK1* is functionally required for E_2_ to modulate key processes implicated in placental detachment: apoptosis, tight junction integrity, and epithelial–mesenchymal transition (EMT). This study aims to provide the first experimental evidence connecting *SGK1* to RP and to investigate the potential role and regulatory relationship of the E_2_-*SGK1* axis using a cellular model, thereby assessing its plausibility as a contributing pathway to this costly disease.

## 2. Materials and Methods

### 2.1. Sample Preparation and Collection

All animal procedures were approved by the Animal Ethics Committee of Gansu Agricultural University (GSAU-Eth-LST-2021-003). This study followed the ARRIVE guidelines, and the completed ARRIVE Essential 10 checklist is provided in Supplementary Document S1. Holstein dairy cows (3–4 years old, parity 2–3, body weight 500 ± 10 kg) were selected from an intensive farm in Zhangye, Gansu Province. All enrolled cows were first sampled and then observed for 12 h to determine their clinical outcome. Based on whether the placenta was expelled spontaneously within 12 h post-calving, cows were retrospectively designated as either normal controls (NC, *n* = 3) or retained placenta cases (RP, *n* = 3).

Peripheral blood samples for RNA-seq were collected from the tail vein within a standardized post-calving window (0.5–3 h). Fetal cotyledonary tissues were collected immediately after spontaneous expulsion in NC cows (within 0.5–3 h post-calving) and following manual removal in RP cows (within 12–24 h post-calving). Cotyledons were dissected from fetal membranes, washed with sterile saline, and then either snap-frozen in liquid nitrogen or fixed in 4% paraformaldehyde. All tissues were processed within 30 min of collection, with care taken to minimize inclusion of maternal caruncular tissue.

### 2.2. Transcriptome Sequencing and Bioinformatics Analysis

The RNA-seq data analyzed in this study were derived from a previously published dataset (GEO accession: GSE214588) generated by our group, comparing postpartum blood transcriptomes between cows with RP and NC. The original methods for sample collection (from NC and RP cows), total RNA extraction using TRIzol reagent (Thermo Fisher Scientific, Waltham, MA, USA), library preparation with the TruSeq Small RNA Sample Prep Kit and Ribo-Zero™ rRNA Removal Kit (Illumina, San Diego, CA, USA), and sequencing have been described in detail therein [29]. For the present study, we re-analyzed this dataset with a specific focus on identifying novel candidate genes involved in hormonal regulation and placental detachment. The functional roles of the DEGs were then investigated through the Metascape platform (https://metascape.org/gp/index.html (accessed on 9 October 2025)), based on Gene Ontology (GO) and KEGG pathway enrichments.

### 2.3. RNA Extraction and Quantitative Real-Time PCR (qPCR)

Total RNA was extracted using TransZol Up reagent (Invitrogen, Carlsbad, CA, USA). After assessing purity and concentration, 500 ng of RNA was reverse-transcribed into cDNA using the Evo M-MLV RT Kit with gDNA Clean (Accurate Biology, Beijing, China). qPCR was performed on a LightCycler 96 System (Roche, Basel, Switzerland) with SYBR Green Premix Pro Taq HS (Accurate Biology, China). Each 20 μL reaction contained 10 μL of 2× Premix, 0.4 μL of each forward and reverse primer (10 μM), 2 μL of cDNA, and 7.2 μL of nuclease-free water. The thermal profile was: 95 °C for 30 s; 40 cycles of 95 °C for 5 s and 60 °C for 30 s. Melt curve analysis (65–95 °C) confirmed amplification specificity.

To control for genomic DNA contamination, a no-reverse transcription (No-RT) control was included for each RNA sample. A no-template control (NTC) was run on every plate. All primers (Supplementary Table S1) were designed to span exon-exon junctions. Amplification efficiency (95–105%) was validated using a standard curve. Gene expression was normalized to *GAPDH* and calculated via the 2^−ΔΔCt^ method. All samples were run in triplicate.

### 2.4. Immunohistochemical Staining

Paraffin sections were deparaffinized and rehydrated with graded ethanol, followed by three washes with phosphate-buffered saline (PBS). Tissue antigens were retrieved by using citrate buffer. Subsequently, immunohistochemical staining was performed using the Histostain™-Plus Kit (Bioss Biotechnology Co., Ltd., Beijing, China). Sections were incubated overnight at 4 °C in a humid chamber with rabbit anti-*SGK1* polyclonal antibody (1:150, 28454-1-AP, Proteintech, Rosemont, IL, USA, Immunohistochemical, AB_2881145). Finally, antigen-antibody binding signals were visualized using a DAB chromogen kit (Bioss Biotechnology Co., Ltd., China), and representative images were observed and captured under an optical microscope (Olympus, Tokyo, Japan). For negative controls, serial adjacent sections were processed in parallel with the primary antibody omitted (replaced with phosphate-buffered saline or non-immune serum). No specific immunostaining was observed in these negative control sections. Representative images of negative controls are shown in Supplementary Figure S2.

### 2.5. Cell Culture, E_2_ Treatment, and Transfection Methods

Given that placental detachment is a process requiring coordinated actions at the fetomaternal interface, and that apoptosis and junctional remodeling of endometrial epithelial cells have been demonstrated to be key initiating events [6,7,9], the bovine endometrial epithelial cell line (BEND) was selected as an initial in vitro model for this study. The BEND cells, originally established from the endometrium of cyclic cows, were maintained by the Gansu Key Laboratory of Animal Generational Physiology and Reproductive Regulation. Cells were routinely cultured at 37 °C in a 5% CO_2_ atmosphere using DMEM/F-12 medium (Gibco, Grand Island, NY, USA), supplemented with 10% fetal bovine serum (Invitrogen, USA) and 1% penicillin/streptomycin (Solarbio, Beijing, China).

The experiment employed E_2_ (C_18_H_24_O_2_, Macklin, Shanghai, China) treatments at different concentrations. The final working concentrations in the culture medium were 7.34 × 10^−10^ mol/L (0.2 ng/mL), 1.84 × 10^−9^ mol/L (0.5 ng/mL), 3.67 × 10^−9^ mol/L (1 ng/mL), 1.84 × 10^−8^ mol/L (5 ng/mL), and 3.67 × 10^−8^ mol/L (10 ng/mL). To prepare these, a stock solution (Solution A) was first made by dissolving 5 mg of E_2_ powder in 5 mL of dimethyl sulfoxide (DMSO), yielding a concentration of 3.67 × 10^−3^ mol/L (1 mg/mL). This solution was sterilized via filtration through a 0.22 μm membrane, aliquoted, and stored at −20 °C. A series of Intermediate stock solutions were then prepared via sequential dilution of Solution A: 3.67 × 10^−5^ mol/L (10 μg/mL), 1.84 × 10^−5^ mol/L (5 μg/mL), 3.67 × 10^−6^ mol/L (1 μg/mL), 1.84 × 10^−6^ mol/L (500 ng/mL), and 7.34 × 10^−7^ mol/L (200 ng/mL). For cell treatment, 2 μL of the corresponding intermediate stock was added to 1998 μL of culture medium, achieving a 1000-fold dilution and the desired final concentration.

In transfection experiments, BEND cells were seeded at approximately 60% confluence in six-well plates. Transfection of si-*SGK1* and the negative control si-NC (Azenta, Suzhou, China), whose sequences are detailed in Supplementary Table S1, was performed using the PepMute™ siRNA transfection reagent (SignaGen Laboratories, Rockville, MD, USA) following the manufacturer’s protocol. The transfection mixture was gently mixed and added dropwise to the wells. Cells were cultured at 37 °C. Fresh medium was replaced 12 h post-transfection, and transfection efficiency was assessed at 24, 48, and 72 h.

### 2.6. Immunofluorescence Staining

Cells were fixed with 4% paraformaldehyde for 30 min, then washed three times with PBS. Subsequently, cells were permeabilized with 0.1% Triton X-100 (Bioss, China) at room temperature for 30 min, followed by three PBS washes. To prevent non-specific binding, the samples were incubated for 30 min at room temperature in a solution of 5% bovine serum albumin (Solarbio, China). After blocking, rabbit anti-*SGK1* polyclonal antibody (1:100, 28454-1-AP, Proteintech, Immunofluorescence, AB_2881145) was added and incubated overnight at 4 °C (≥10 h). Following primary antibody incubation, samples were incubated with an Alexa Fluor^®^ 488-conjugated goat anti-rabbit secondary antibody at 37 °C for 1 h in the dark. Finally, DAPI was applied to counterstain the cell nuclei.

### 2.7. Western Blotting

Western blot analysis was conducted as follows. Total protein extracts from fetal cotyledons or cultured cells were prepared using RIPA lysis buffer (Solarbio, China), supplemented with PMSF to inhibit proteases. Protein concentrations were determined with a BCA assay kit (Solarbio, China). Subsequently, protein samples were combined with loading buffer and heat-denatured at 100 °C for 10 min. Equal amounts of protein (30 μg per lane) were subjected to electrophoretic separation on 12% SDS-polyacrylamide gels and then electrotransferred onto PVDF membranes (Millipore, Burlington, MA, USA). Membranes were blocked with 5% non-fat milk in TBST for 1 h at room temperature and then incubated with the indicated primary antibodies diluted in blocking buffer overnight at 4 °C with gentle shaking. Primary antibodies and their dilutions included GAPDH (Mouse monoclonal, 1:5000, 60004-1-Ig, Proteintech, WB, AB_2107436), *SGK1* (Rabbit polyclonal, 1:1000, 28454-1-AP, Proteintech, WB, AB_2881145), BAX (Rabbit monoclonal, 1:3000, 50599-2-Ig, Proteintech, WB, AB_2061561), Caspase-3 (Rabbit monoclonal, 1:1000, TA6311, Abmart, WB, AB_3717821), Bcl2 (Rabbit monoclonal, 1:1000, T40056, Abmart, Shanghai, China, WB, AB_2929011), ZO1 (Rabbit polyclonal, 1:2000, 21773-1-AP, Proteintech, WB, AB_10733242), Occludin (Rabbit polyclonal, 1:2000, 27260-AP, Proteintech, WB, AB_2880820), E-cadherin (Rabbit monoclonal, 1:1500, 20874-1-AP, Proteintech, WB, AB_10697811), and N-cadherin (Rabbit polyclonal, 1:2000, 22018-1-AP, Proteintech, WB, AB_2813891).

The membranes were then washed three times with PBST and incubated with the corresponding secondary antibodies (Goat Anti-Rabbit IgG, SA00001-2, 1:5000 or Goat anti-Mouse IgG, SA00001-1, 1:5000; Proteintech) at 37 °C for 2 h. Finally, the signal was visualized using an ECL chemiluminescent kit (Abnova, Taipei, China) and captured with a chemiluminescence imaging system. The gray values of protein bands were quantified using ImageJ 1.48 (NIH, Bethesda, MD, USA) software.

For negative controls to assess antibody specificity, membranes were incubated under identical conditions with normal rabbit IgG or normal mouse IgG at the same protein concentration as the corresponding primary antibody. The original, uncropped images of all Western blots are provided in Supplementary Figure S1, and representative images of negative controls are shown in Supplementary Figure S2.

### 2.8. Data Statistics and Analysis

All data were analyzed using GraphPad Prism 9 and are presented as mean ± SD from three independent replicates. Normality (Shapiro–Wilk test) and homogeneity of variances (Levene’s test) were assessed; all data met parametric assumptions (*p* > 0.05). Intergroup comparisons were performed using *t*-tests (two groups) or one-way ANOVA with Tukey’s post hoc test (multiple groups). Statistical significance was set at * *p* < 0.05 and ** *p* < 0.01. Exact *p*-values are available from the corresponding author upon request. Effect sizes (Cohen’s d) with 95% confidence intervals were calculated for pairwise comparisons.

## 3. Results

### 3.1. Bioinformatic Prioritization of SGK1 as a Candidate Gene from Blood Transcriptome Data

**Figure 1:**
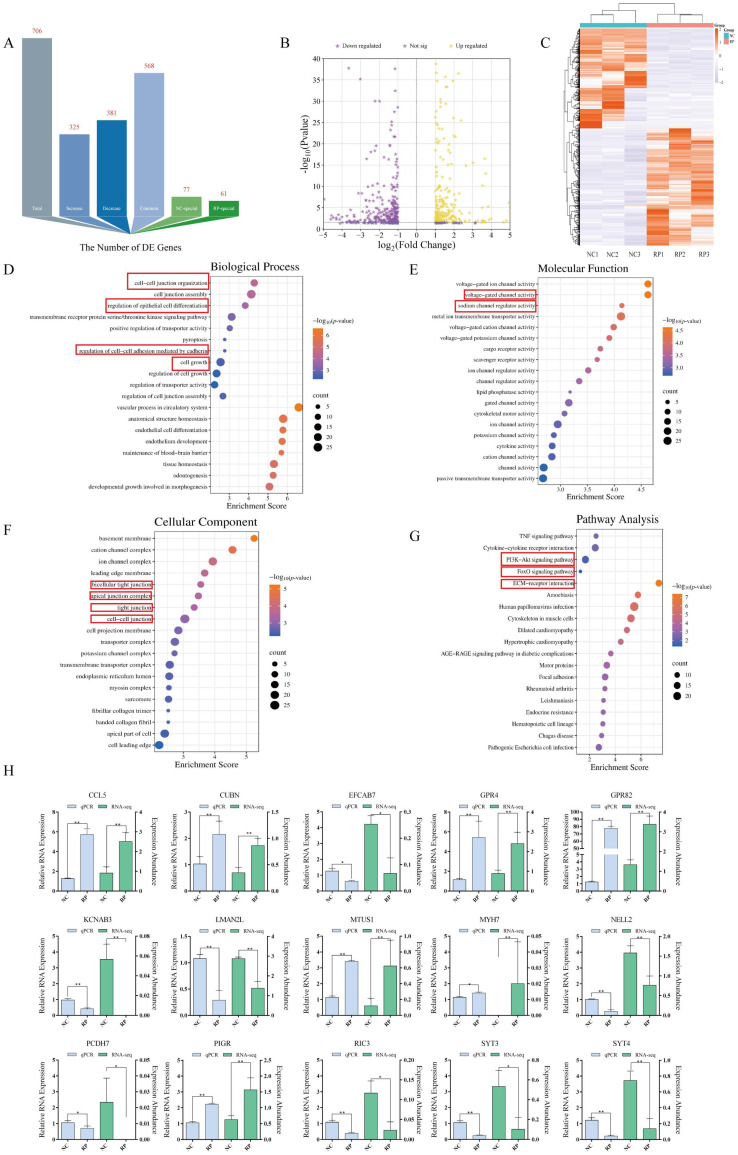
mRNA sequencing analysis and validation of differentially expressed genes (DEGs). (**A**) Statistics on the number of differentially expressed genes. (**B**) Volcano plot of differentially expressed genes. (**C**) Heatmap of expression patterns for differentially expressed genes. (**D**) GO functional enrichment analysis of differentially expressed genes: Biological Process. (**E**) GO functional enrichment analysis of differentially expressed genes: Molecular Function. (**F**) GO functional enrichment analysis of differentially expressed genes: Cellular Component. (**G**) KEGG pathway enrichment analysis of differentially expressed genes. Key biological processes and signaling pathways are highlighted with red rectangles. Pathways and biological processes of particular interest to this study are highlighted with red rectangles. (**H**) qPCR validation of expression levels for differentially expressed genes. * *p* < 0.05, ** *p* < 0.01.

RNA sequencing analysis of postpartum blood revealed a distinct transcriptomic profile associated with RP, identifying 706 differentially expressed genes (DEGs) compared to normal controls (NC) (Figure 1A–C). Validation by qPCR confirmed the reliability of this dataset for 15 randomly selected DEGs (Figure 1H).

Gene Ontology (GO) enrichment analysis indicated these DEGs were significantly overrepresented in biological processes critical for tissue detachment, including cell-cell junction assembly, epithelial cell differentiation, and hormone response (Figure 1D–F). KEGG pathway analysis further highlighted enrichment in the PI3K-Akt and FoxO signaling pathways (Figure 1G), which are central to regulating cell survival, metabolism, and adhesion.

**Figure 2:**
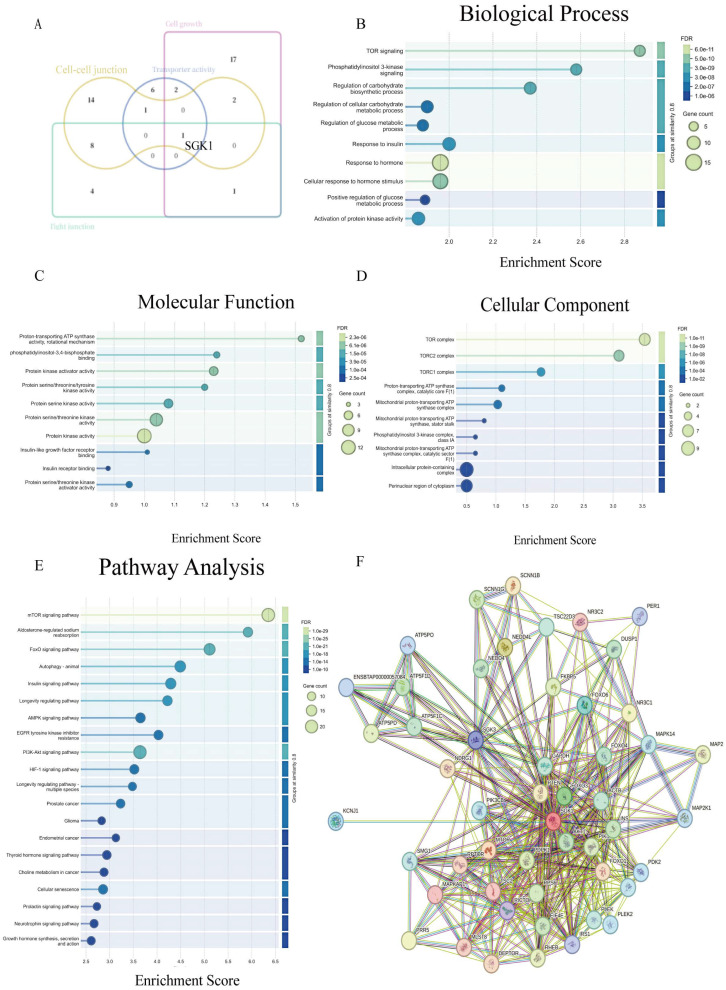
Screening and functional analysis of Serum and Glucocorticoid-regulated Kinase 1 (*SGK1*). (**A**) Venn diagram identifying the differentially expressed gene *SGK1*. (**B**) GO functional enrichment analysis of *SGK1*: Biological Process. (**C**) GO functional enrichment analysis of *SGK1*: Molecular Function. (**D**) GO functional enrichment analysis of *SGK1*: Cellular Component. (**E**) KEGG pathway enrichment analysis of *SGK1*. (**F**) Protein–protein interaction (PPI) network.

To generate testable hypotheses, we cross-referenced blood-derived DEGs with biological processes relevant to placental detachment. *SGK1* was prioritized as a candidate for subsequent tissue validation (Figure 2A). Independent functional annotation of *SGK1* confirmed its strong association with hormone response and the PI3K-Akt signaling pathway (Figure 2B–E). Protein–protein interaction (PPI) network analysis further positioned *SGK1* as a central node involved in regulating cellular stress responses and survival processes (Figure 2F). Based on this convergent evidence from differential expression, functional enrichment, and network topology, *SGK1* was selected as the lead candidate for direct examination in placental tissues. In summary, *SGK1* was prioritized as a candidate gene not merely due to its differential expression in blood, but because its functional profile strongly implicated it in the biological processes governing tissue adhesion and detachment. This warranted its further investigation in the local placental context.

### 3.2. SGK1 Expression Is Elevated in RP Fetal Cotyledonary Tissues in a Preliminary Cohort

**Figure 3:**
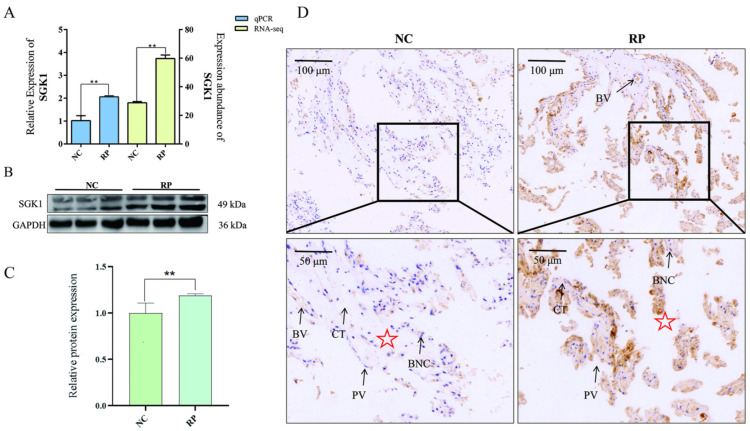
Expression analysis of *SGK1* in fetal cotyledonary tissues from NC and RP cows. (**A**) qPCR detection of *SGK1* gene expression, consistent with RNA-seq results. Values represent mean ± SD, *n* = 3, ** *p* < 0.01. (**B**) Western blot detection of *SGK1* protein expression levels. (**C**) Gray-scale analysis of Western blot results. Values represent mean ± SD, *n* = 3, ** *p* < 0.01. (**D**) Immunohistochemical detection of *SGK1* expression in different tissues. CT: Connective tissue; BV: Blood vessel; PV: Placental villi; BNC: Binucleated trophoblast cell; **✰**: Placental villi fragmentation comparison.

To assess the association of *SGK1* with RP, we analyzed its expression in fetal cotyledons. Relative to the NC group, *SGK1* mRNA levels were significantly elevated in the RP group (Figure 3A). Protein analysis by Western blot confirmed this trend, showing augmented *SGK1* protein expression in RP tissues (Figure 3B,C). Immunohistochemistry further revealed stronger *SGK1* immunostaining, with particularly intense signals in the trophoblast cells of RP tissues compared to NC (Figure 3D). Together, these data from a limited sample set preliminarily associate elevated *SGK1* levels with the RP condition.

### 3.3. Apoptosis Is Attenuated in RP Fetal Cotyledonary Tissues

**Figure 4:**
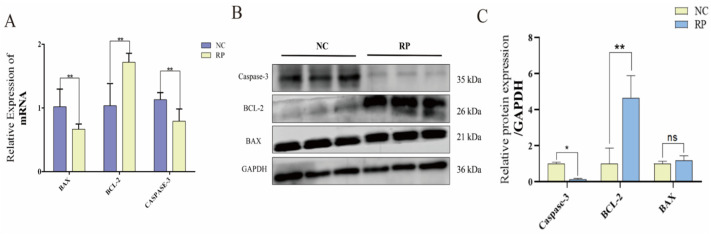
Expression analysis of apoptosis markers in fetal cotyledonary tissues from NC and RP cows. (**A**) qPCR detection of apoptosis gene expression. Values represent mean ± SD, *n* = 3. ** *p* < 0.01. (**B**) Western blot detection of apoptosis-related protein expression levels. (**C**) Gray-scale analysis of WB results. Values represent mean ± SD, *n* = 3. ns, not significant; * *p* < 0.05, ** *p* < 0.01.

We evaluated the expression of apoptosis-related markers in fetal cotyledonary tissues. Compared to NC tissues, RP tissues exhibited significantly downregulated Caspase-3 at both mRNA and protein levels. While *BAX* mRNA was decreased, its protein level showed no significant change. In contrast, the anti-apoptotic protein BCL-2 was significantly upregulated at both transcriptional and translational levels in RP tissues (Figure 4A–C). Consequently, the Bax/Bcl-2 protein ratio and Caspase-3 activity were reduced in RP tissues, collectively indicating a state of attenuated apoptotic activity associated with RP in this initial tissue set.

### 3.4. Altered Expression of Tight Junction and EMT-Associated Markers in RP Fetal Cotyledonary Tissues

**Figure 5:**
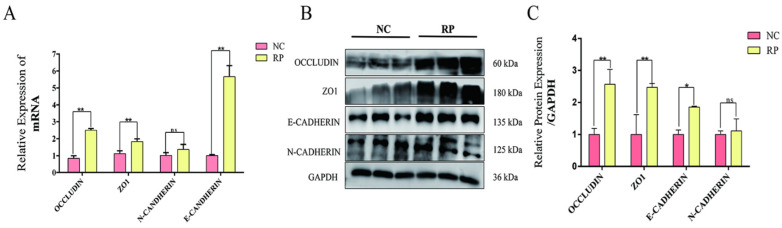
Expression analysis of tight junction-associated and EMT-related proteins in fetal cotyledonary tissues from NC and RP cows. (**A**) qPCR detection of tight junction and EMT-related gene expression. Values represent mean ± SD, *n* = 3. ns, not significant; ** *p* < 0.01. (**B**) Western blot detection of tight junction and EMT-related protein expression levels. (**C**) Gray-scale analysis of Western blot results. Values represent mean ± SD, *n* = 3. ns, not significant; * *p* < 0.05, ** *p* < 0.01.

Analysis of tight junction and EMT markers revealed distinct expression profiles in RP tissues. Both mRNA and protein levels of the tight junction-associated proteins *ZO-1* and *Occludin* were notably upregulated in the RP group compared to the NC group (Figure 5A–C). For EMT markers, the epithelial marker E-cadherin was significantly elevated at both mRNA and protein levels in RP tissue. The mRNA level of the mesenchymal marker *N-cadherin* showed an upward trend without statistical significance, and its protein expression remained unchanged (Figure 5A–C). The resulting significant increase in the E-cadherin/N-cadherin expression ratio is consistent with a shift toward a more epithelial phenotype, which may reflect an altered cellular state in this preliminary cohort.

### 3.5. Optimization of E_2_ Treatment and SGK1 Knockdown in BEND Cells

**Figure 6:**
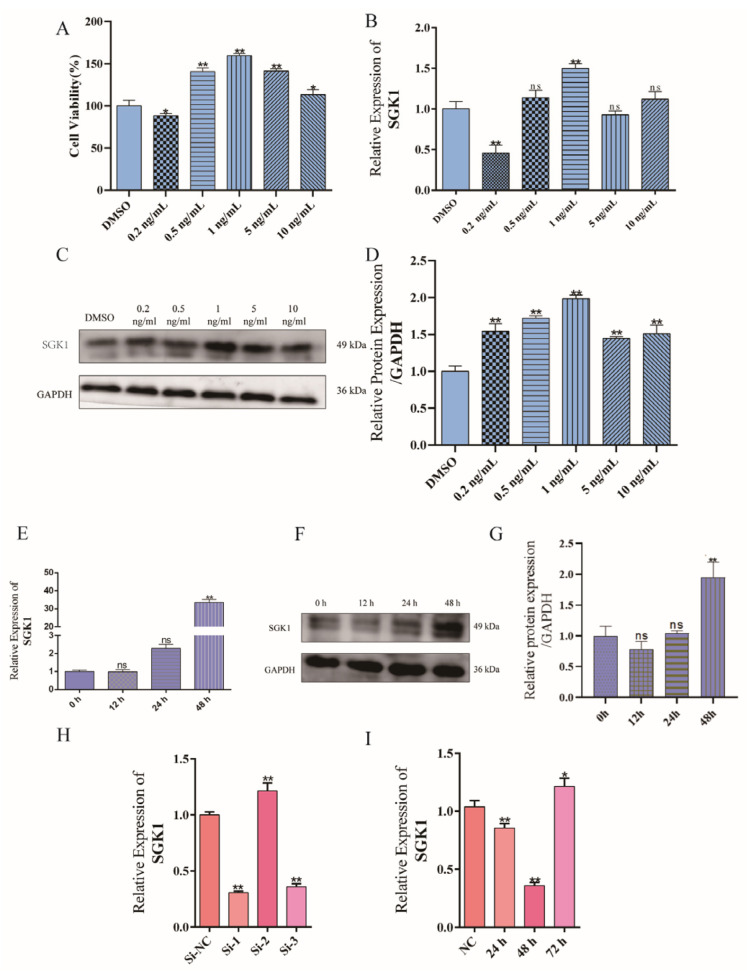
Screening of estradiol (E_2_) treatment conditions and validation of *SGK1* knockdown efficiency. (**A**) CCK-8 assay to detect the effects of different E_2_ concentrations on cell viability, * *p* < 0.05; ** *p* < 0.01; (**B**) qPCR analysis of *SGK1* mRNA expression levels under different E_2_ concentrations. Values represent mean ± SD, *n* = 3. ns, not significant; ** *p* < 0.01; (**C**) Western blot detection of *SGK1* protein expression under different E_2_ concentrations; (**D**) Statistical analysis of gray values from Western blot results. Values represent mean ± SD, *n* = 3. ** *p* < 0.01; (**E**) qPCR detection of *SGK1* mRNA expression after 12, 24, and 48 h of 1 ng/mL E_2_ treatment. Values represent mean ± SD, *n* = 3. ns, not significant; ** *p* < 0.01; (**F**) Western blot detection of *SGK1* protein expression after 12, 24, and 48 h of 1 ng/mL E_2_ treatment; (**G**) Statistical analysis of gray values. Values represent mean ± SD, *n* = 3. ns, not significant; ** *p* < 0.01; (**H**) qPCR screening for knockdown efficiency of different si-*SGK1* sequences, ** *p* < 0.01; (**I**) qPCR detection of *SGK1* knockdown efficiency at 24, 48, and 72 h post-transfection with si-*SGK1*-1. Values represent mean ± SD, *n* = 3. * *p* < 0.05, ** *p* < 0.01.

To establish an in vitro model for investigating the E_2_-*SGK1* axis, we first determined the optimal treatment conditions in bovine endometrial epithelial (BEND) cells. CCK-8 assays indicated that E_2_ promoted cell proliferation (Figure 6A). E_2_ treatment upregulated *SGK1* expression in a concentration- and time-dependent manner at both mRNA and protein levels (Figure 6B–D). Based on these results, 1 ng/mL E_2_ treatment for 48 h was selected for subsequent experiments, as it induced peak *SGK1* expression (Figure 6E–G). For functional intervention, we screened siRNA sequences and found si-*SGK1*-1 achieved the most potent knockdown efficiency, optimally at 48 h post-transfection (Figure 6H,I).

### 3.6. E_2_ Upregulates SGK1 Expression in BEND Cells

**Figure 7:**
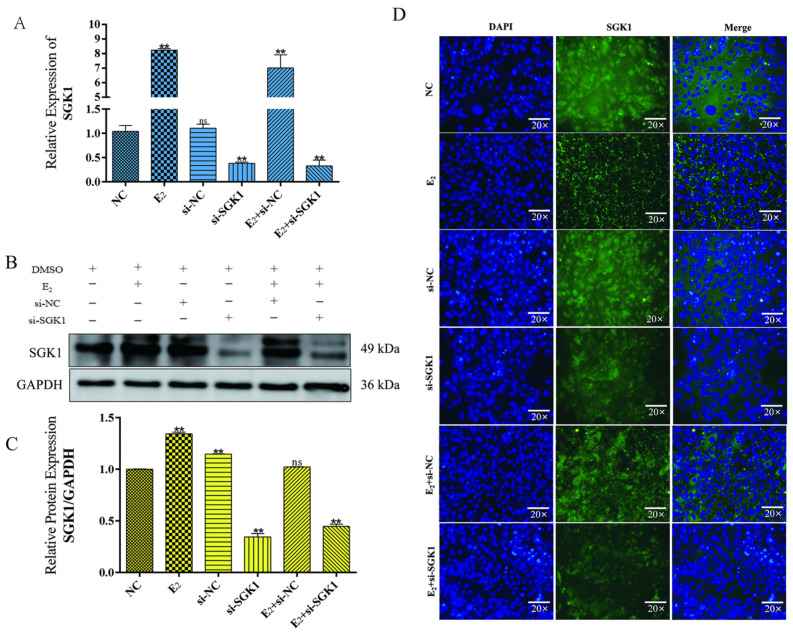
Regulatory effects of E_2_ and *SGK1* knockdown on *SGK1* expression. (**A**) qPCR detection of *SGK1* mRNA expression levels under different treatment conditions. Values represent mean ± SD, *n* = 3. ns, not significant; ** *p* < 0.01; (**B**) Western blot analysis of *SGK1* protein expression under different treatment conditions; (**C**) Statistical analysis of gray values represent mean ± SD, *n* = 3. ns, not significant; ** *p* < 0.01; (**D**) Immunofluorescence observation of *SGK1* protein expression and localization under different treatment conditions.

Having established the model, we systematically compared six experimental groups: NC, E_2_, si-NC, si-*SGK1*, E_2_ + si-NC, and E_2_ + si-*SGK1*. qPCR and Western blot analyses confirmed that E_2_ treatment significantly elevated *SGK1* expression, while *SGK1* knockdown effectively reduced its expression both in the absence and presence of E_2_ (Figure 7A–C). Immunofluorescence staining corroborated these findings, showing enhanced *SGK1* (green) fluorescence intensity upon E_2_ treatment and markedly diminished signal after *SGK1* knockdown (Figure 7D).

### 3.7. SGK1 Mediates the Anti-Apoptotic Effect of E_2_ in BEND Cells

**Figure 8:**
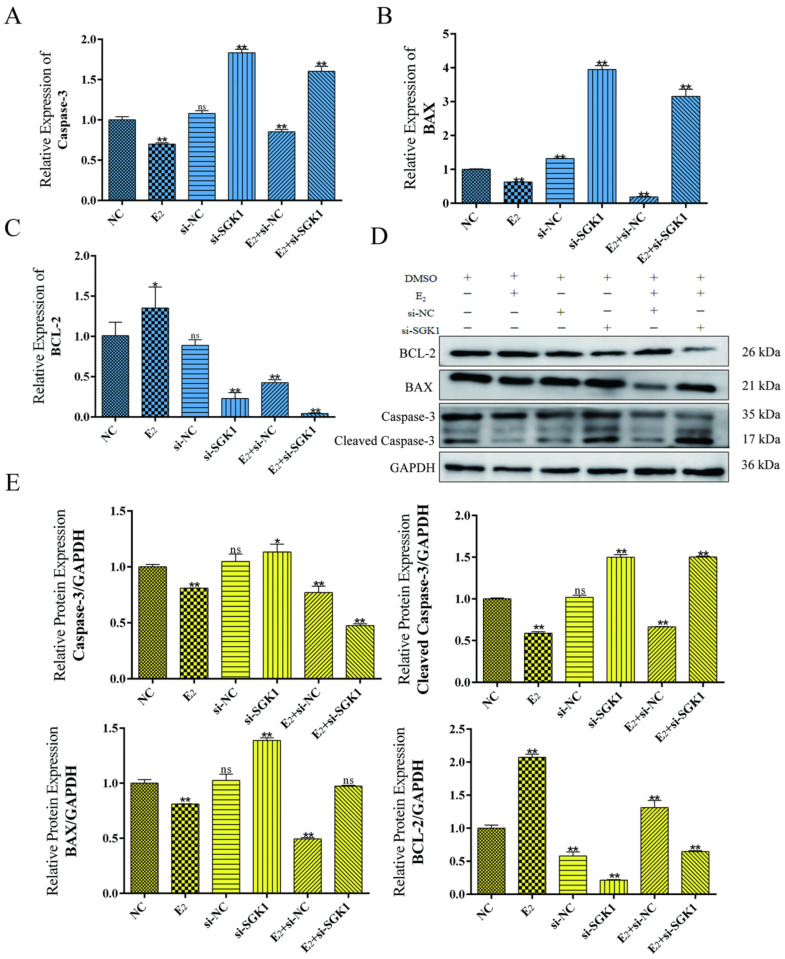
Effects of E_2_ and *SGK1* knockdown on apoptosis-related markers. (**A**–**C**) qPCR detection of *Caspase3*, *BAX*, and *BCL-2* mRNA expression levels under different treatment conditions. Values represent mean ± SD, *n* = 3. ns, not significant; * *p* < 0.05, ** *p* < 0.01; (**D**) Western blot analysis of Cleaved Caspase-3, Caspase3, BAX, and BCL-2 protein expression under different treatment conditions; (**E**) Statistical analysis of gray values for Western blot results. Values represent mean ± SD, *n* = 3. ns, not significant; * *p* < 0.05, ** *p* < 0.01.

In the BEND cell model, E_2_ treatment significantly reduced pro-apoptotic indicators (BAX, Caspase-3, Cleaved Caspase-3) and elevated the anti-apoptotic protein BCL-2 compared to the control (Figure 8A–E). Conversely, *SGK1* knockdown alone increased pro-apoptotic markers and decreased BCL-2, promoting apoptosis. Importantly, in cells with *SGK1* knockdown (E_2_ + si-*SGK1* group), E_2_ treatment failed to reverse this pro-apoptotic phenotype; apoptosis levels remained high, as indicated by an elevated Bax/Bcl-2 ratio and Cleaved Caspase-3 level (Figure 8D,E).

### 3.8. SGK1 Is Required for E_2_-Modulated Expression of Tight Junction Proteins, EMT-Associated Marker Changes, and Migration in BEND Cells

**Figure 9:**
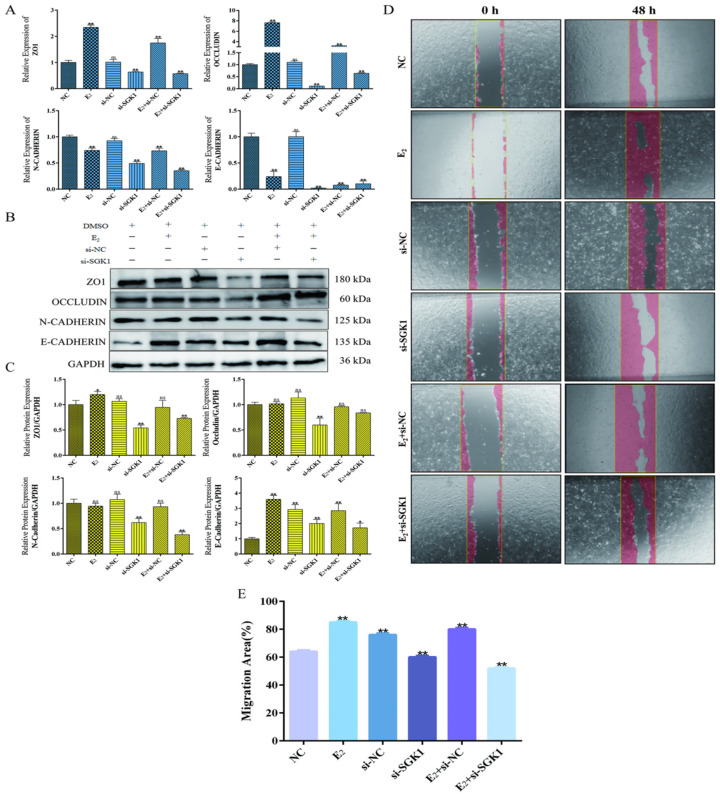
Effects of E_2_ and *SGK1* knockdown on tight junction protein expression, EMT-associated marker changes, and cell migration. (**A**) qPCR detection of mRNA expression levels of *ZO-1*, *Occludin*, *E-cadherin*, and *N-cadherin* under different treatment conditions. Values represent mean ± SD, *n* = 3. ns, not significant; ** *p* < 0.01; (**B**) Western blot analysis of protein expression of ZO-1, Occludin, E-cadherin, and N-cadherin under different treatment conditions; (**C**) Statistical analysis of grayscale values. Values represent mean ± SD, *n* = 3. ns, not significant; * *p* < 0.05, ** *p* < 0.01; (**D**) Cell scratch assay evaluating the effects of different treatments on cell migration capacity; (**E**) Quantitative analysis of migration rates. Values represent mean ± SD, *n* = 3, ** *p* < 0.01.

E_2_ treatment alone upregulated the mRNA levels of tight junction proteins *ZO-1* and *Occludin*, a trend that was not fully recapitulated at the protein level under these conditions. *SGK1* knockdown significantly reduced both mRNA and protein levels of ZO-1 and Occludin. Interestingly, in *SGK1*-knockdown cells, E_2_ co-treatment partially restored the expression of these tight junction proteins (Figure 9A–C), suggesting the involvement of additional, *SGK1*-independent pathways in E_2_-mediated regulation of these junctional components.

Regarding the expression of EMT-associated markers, E_2_ treatment increased E-cadherin protein and the E-cadherin/N-cadherin ratio, while *SGK1* knockdown led to a complex change, elevating E-cadherin but decreasing N-cadherin protein (Figure 9A–C). Wound healing assays demonstrated that E_2_ promoted cell migration, whereas *SGK1* knockdown markedly impaired it. Critically, the pro-migratory effect of E_2_ was abolished in SGK1-knockdown cells (E_2_ + si-*SGK1*), as migration rates remained low (Figure 9D,E).

## 4. Discussion

Our study provides the first evidence implicating *SGK1* in the RP in dairy cows. We demonstrate that *SGK1* is significantly upregulated in RP fetal cotyledons in our preliminary cohort, and that this elevation is correlated with a distinct cellular phenotype characterized by suppressed apoptosis, increased expression of tight junction-associated proteins, and an inhibition of the EMT. Importantly, this model is derived from a sequential investigative approach: bioinformatic screening of system-level (blood) transcriptomic data identified *SGK1* as a candidate functionally linked to relevant pathways; this candidacy was then confirmed by its marked upregulation within the RP fetal cotyledon tissue itself; finally, its functional role as a mediator of E_2_ signaling was established in a reductionist cellular model.

**Figure 10:**
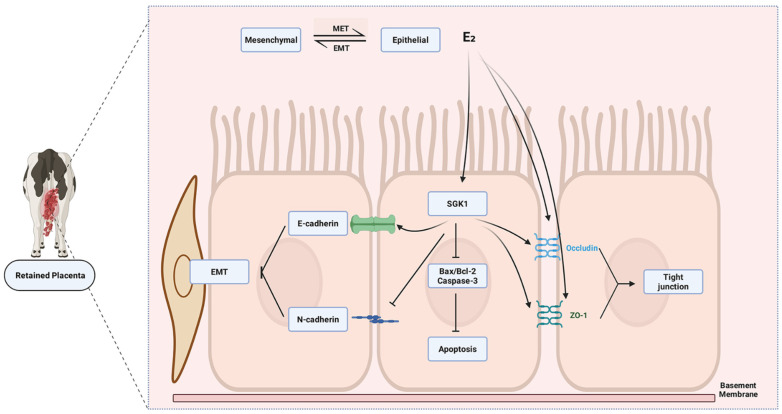
A Proposed Working Model: The Potential Role of the E_2_-*SGK1* Axis in RP. This schematic summarizes a working hypothesis based on the data from this study. In BEND cells, we demonstrated that E_2_ upregulates *SGK1* and that *SGK1* mediates the effects of E_2_ on apoptosis, tight junction protein expression, and cell migration/EMT-like phenotype. Integrating these in vitro mechanistic findings with the observed upregulation of *SGK1* and a correlated hyper-stabilized cellular phenotype in RP fetal cotyledonary tissues from a preliminary cohort, we propose a model whereby dysregulated periparturient E_2_ signaling may lead to sustained *SGK1* activation. This could promote excessive stabilization of the fetomaternal interface, potentially contributing to the failure of timely placental detachment.

Based on this convergence of tissue-level association and in vitro mechanistic data, we put forward a novel working model: the dysregulation of a periparturient “E_2_-*SGK1*” axis might represent a potential pathway contributing to RP by promoting excessive cellular stabilization at the fetomaternal interface, thereby physically impeding the programmed tissue separation that is essential for normal placental expulsion (Figure 10).

### 4.1. SGK1: From a Guardian of Pregnancy to a Potential Pathological Factor in Parturition

*SGK1* is well-established as a critical mediator of endometrial receptivity and embryo implantation in humans and rodents, primarily acting as a downstream effector of progesterone to support decidualization and cell survival [22,28]. Its function is finely tuned like a “fertility switch”: its expression must be transiently downregulated during the implantation window to permit embryo adhesion, yet restored thereafter to maintain uterine quiescence [30,31]. Our study extends the relevance of *SGK1* to bovine reproduction and reveals a crucial functional paradox: this vital “guardian” of early pregnancy may, through its sustained and aberrant overexpression during the periparturient period, transform into an “obstacle” contributing to RP.

This functional shift of *SGK1* from a putative “pregnancy establisher” to a potential “parturition obstructer” critically depends on its temporal and context-specific regulation. Successful placental detachment requires precisely coordinated cellular events, including apoptosis initiation, tight junction disassembly, and a carefully regulated degree of EMT to enable cellular motility and tissue separation. Our data reveal that sustained high *SGK1* expression in RP placentas correlates with a “hyper-stabilized” cellular state, characterized by suppressed apoptosis, increased expression of tight junction proteins, and an inhibition of the EMT process—the latter evidenced by an elevated *E-cadherin/N-cadherin* ratio. This phenotype aligns with and extends findings from studies such as those on vimentin expression in bovine placenta, where impaired or dysregulated EMT has been associated with placental retention [32]. Together, these observations suggest that *SGK1* may act as an important regulator that synchronously reinforces the fetomaternal interface, thereby physically impeding the programmed separation cascade. The in vitro evidence that E_2_ upregulates *SGK1* and that *SGK1* knockdown abolishes key E_2_-mediated effects provides direct mechanistic plausibility for the pathogenic role of a dysregulated “E_2_-*SGK1*” axis in RP.

Our findings resonate with and extend insights from other reproductive pathologies, painting a broader picture of *SGK1* dysregulation. In human studies, excessively high *SGK1* expression has been linked to impaired endometrial receptivity and infertility [33,34,35], while insufficient levels are associated with an increased risk of recurrent pregnancy loss [36,37]. More instructively, research on “placental premature aging” has found significantly elevated *SGK1* expression in pathological fetal cotyledonary tissues, correlating with the upregulation of the pro-fibrotic factor CTGF and marked tissue fibrosis. This implies that aberrant *SGK1* activation may not only inhibit normal tissue remodeling but could also drive pathological fibrotic processes. These two mechanisms may synergistically contribute to the loss of fetal cotyledonary tissues’ elasticity and separation failure. Recent epigenetic studies further indicate that *SGK1* is a key target during the gene reprogramming of pre-implantation uterine luminal epithelium, and its timely silencing is crucial for the transition to a receptive state [38]. This underscores, from another perspective, that once the precise “temporal window” of *SGK1* expression is disrupted, it may derail the entire subsequent chain of reproductive events.

### 4.2. The Paradoxical Phenotype: SGK1’s Dual Potential in Stabilization Versus Migration

A particularly intriguing and seemingly paradoxical finding of our study is that elevated *SGK1* expression in RP is associated with a stabilized cellular phenotype, characterized by the inhibition of EMT and the reinforcement of intercellular junctions. This stands in direct contrast to its well-established role in diverse carcinomas, where *SGK1* acts as a potent driver of EMT, cell migration, and metastasis [39]. This stark functional duality underscores that *SGK1* is not a linear regulator with a fixed outcome, but rather a highly context-dependent signaling node whose biological output is dictated by the integrative signals of its specific microenvironment.

This apparent paradox highlights the remarkable functional plasticity of *SGK1*, whose role is fundamentally reprogrammed by the microenvironment to meet opposing physiological demands: promoting detachment and migration in one context, and enforcing adhesion and integrity in another. In cancer, *SGK1* is often activated by growth factors and survival signals within a hypoxic and inflammatory stroma, co-opting its pro-survival functions to fuel invasion and dissemination [40,41,42]. In contrast, the bovine placental microenvironment at term is dominated by a precise, sharp shift in steroid hormones—notably a pre-partum E_2_ surge—alongside unique inflammatory and mechanical cues [43,44]. We propose that the sustained activation of the E_2_-*SGK1* axis beyond parturition pathologically extends this physiological “stabilization program”. Instead of being transiently activated to manage tissue stress, persistent *SGK1* signaling chronically reinforces tight junctions, suppresses apoptotic and EMT-related detachment signals, and thereby “locks” the fetomaternal interface in a state of abnormal adhesion.

### 4.3. The E_2_–SGK1 Axis: A Previously Unrecognized Hormonal Signaling Link in Parturition

While *SGK1* is a recognized target of progesterone [45], our in vitro data robustly show that it is also a sensitive and direct target of E_2_ in bovine endometrial cells. This identifies a previously unexplored endocrine arm in the regulation of *SGK1* during the peri-parturient period. The abnormal prepartum E_2_ surge, a known risk factor for RP, could thus exert its detrimental effects, at least in part, through sustained *SGK1* activation. Our rescue experiments, where E_2_ failed to reverse the pro-apoptotic and anti-migratory phenotypes in *SGK1*-knockdown cells, strongly argue that *SGK1* is a key mediator of these specific E_2_ effects in this cellular model. The partial rescue of tight junction proteins, however, hints at the existence of additional, parallel pathways by which E_2_ can influence intercellular adhesion, warranting further investigation.

Our study focused on E_2_ as a regulator of *SGK1* based on the well-documented aberrant E_2_ surge in RP. However, we acknowledge that *SGK1* expression is also directly regulated by glucocorticoids and progesterone. The periparturient rise in fetal cortisol is the primary trigger for placental progesterone metabolism and the prepartum E_2_ surge; thus, upstream dysregulation in cortisol signaling or a delayed decline in progesterone could concurrently affect *SGK1* expression and contribute to the RP phenotype. Moreover, successful placental expulsion requires not only detachment of the fetomaternal interface but also adequate uterine contractility driven by oxytocin (OT) and prostaglandins (PGF_2_α). Estradiol is known to prime the uterus for parturition by upregulating endometrial oxytocin receptors and stimulating prostaglandin synthesis [46], and cows with RP exhibit significantly lower concentrations of OT, oxytocin receptors, and PGF_2_α in placental tissues [47]. Whether the E_2_-*SGK1* axis we have identified interacts with this OT-PGF_2_α pathway—for example, through *SGK1*-mediated regulation of oxytocin receptor or COX-2 expression—remains unknown and represents an important direction for future investigation. The absence of longitudinal hormonal profiles (cortisol, progesterone, E_2_) in our cohort is a limitation. Future studies should correlate *SGK1* expression with serial periparturient hormone measurements and examine key markers such as *NR3C1* (glucocorticoid receptor), *CYP19A1* (aromatase), *OXTR*, and *PTGS2/COX-2* in RP versus normal fetal cotyledonary tissues to clarify the integrative endocrine dysregulation underlying RP.

### 4.4. Study Limitations and Future Perspectives

While our study provides novel insights using a well-defined in vitro model, it is important to acknowledge its constraints to contextualize the findings. The primary limitations of this study are its small clinical sample size and the correlative nature of the evidence. First, the small sample size (*n* = 3 per group) may limit the generalizability of the tissue-level findings, and validation in a larger, independent cohort is required. Second, the lack of continuous periparturient hormone profiles in the study cohort precludes the direct establishment of an individual-level correlation between E_2_ and placental *SGK1*. Therefore, the proposed role of the E_2_-*SGK1* axis should be regarded as a working model that integrates established clinical knowledge (E_2_ dysregulation in RP) with our novel correlative and in vitro mechanistic data. Finally, the mechanistic findings are derived from a monolayer endometrial epithelial cell model, which, while valuable for delineating cell-autonomous pathways, cannot fully recapitulate the complex in vivo fetomaternal interface. Future studies employing larger cohorts with hormone profiling, alongside more physiologically relevant models (e.g., tissue explants, in vivo approaches), are essential to confirm the pathophysiological relevance and causal role of this axis in RP.

It is important to note that while our in vitro data demonstrate *SGK1*-mediated E_2_ effects on tight junction protein expression in BEND cells—and such upregulation is widely accepted as correlating with enhanced barrier function [48,49], definitive morphological evidence of junctional reinforcement and in vivo causal evidence for the E_2_-*SGK1* axis in RP pathogenesis remain to be established. Future studies employing complementary approaches (immunohistochemistry, electron microscopy, and animal models) are required to confirm these findings.

## 5. Conclusions

In conclusion, this study preliminarily identifies *SGK1* as a novel molecular factor whose expression is associated with RP in dairy cows. We provide the first experimental evidence that, in a bovine endometrial cell model, *SGK1* acts as a mediator of E_2_ effects on key cellular processes—apoptosis, the expression of junctional proteins, and the regulation of EMT-associated markers. The definitive in vivo role and causal contribution of the E_2_-*SGK1* axis in RP remain to be established. However, our work provides a robust foundational hypothesis and a clear cellular mechanistic framework for future investigation.

The primary significance of this study is scientific: it shifts the conceptual focus from systemic endocrine dysfunction to dysregulation of cellular stabilization programs at the fetomaternal interface, opening a new avenue for understanding RP pathophysiology. The consistent upregulation of *SGK1* in RP placental tissues also positions it as a preliminary candidate biomarker, but this translational potential requires rigorous validation in large, independent cohorts before any application in risk prediction or targeted prevention can be considered.

## Data Availability

The RNA-seq data analyzed in this study are available from the GEO database under accession number GSE214588, a dataset previously published by our group. All additional data generated or analyzed during the current study are included in this article and its Supplementary Information files. Further inquiries can be directed to the corresponding author(s).

## Supplementary Materials

The following supporting information can be downloaded at https://www.mdpi.com/article/10.3390/cells15060558/s1. Table S1: Information of primer sequences for PCR and si-RNA sequences. Figure S1: Original, uncropped Western blot images. Figure S2: Representative negative controls for Western blot and immunohistochemistry. Document S1: ARRIVE Essential 10 author checklist.

## Abbreviations

The following abbreviations are used in this manuscript:

RP	Retained placenta
E_2_	Estradiol
*SGK1*	Serum and Glucocorticoid-Regulated Kinase 1
DEGs	Differentially expressed genes
GO	Gene Ontology
KEGG	Kyoto Encyclopedia of Genes and Genomes
PPI	Protein–protein interaction
BEND	Bovine endometrial epithelial cell line
EMT	Epithelial–mesenchymal transition
