# Supplementary material for: SGK1 Is Upregulated in Retained Placenta and Mediates Estradiol Effects in Bovine Endometrial Cells
Source: Cells. 2026 Mar 20;15(6):558. doi: 10.3390/cells15060558 (PMC13025382; doi:10.3390/cells15060558)

FIG.3 SGK1

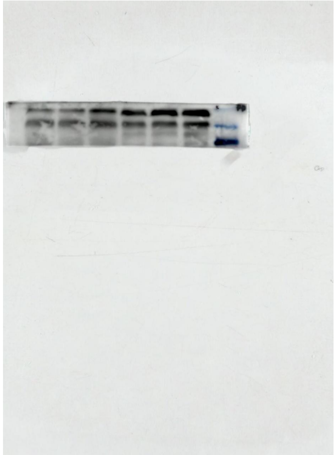

FIG.3 GAPDH

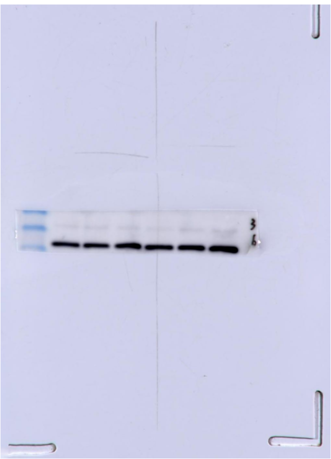

FIG.3 NC 20X

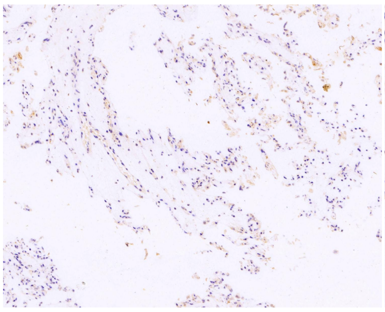

NC 40X

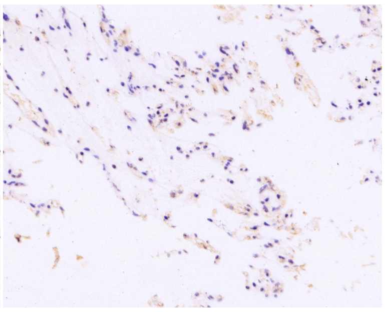

FIG.3 RP 20X

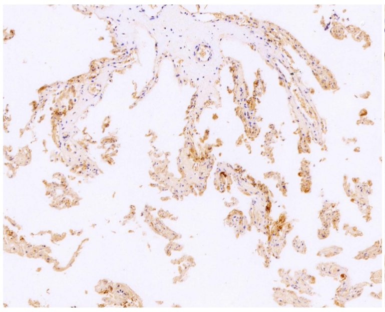

RP 40X

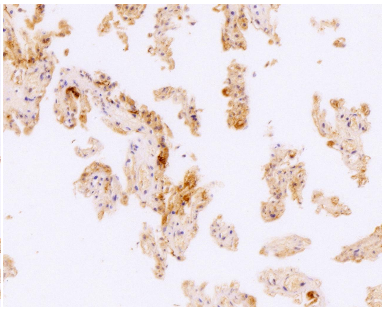

FIG.4 BAX

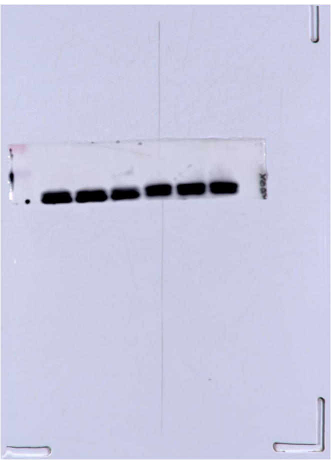

FIG.4 GAPDH

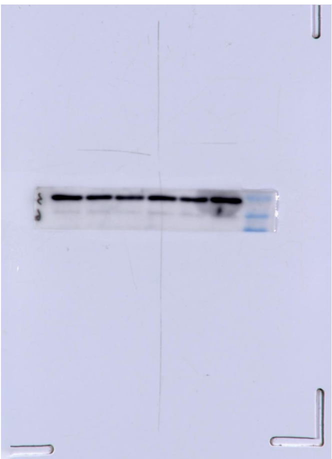

FIG.4 BCL-2

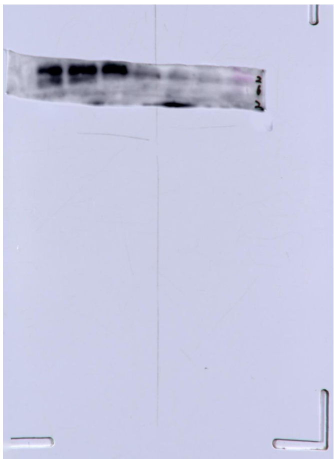

FIG.4 Caspase-3

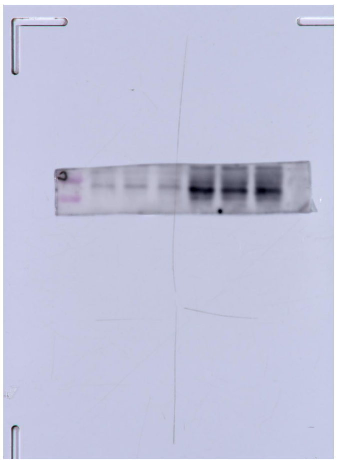

FIG.5 E-cadherin

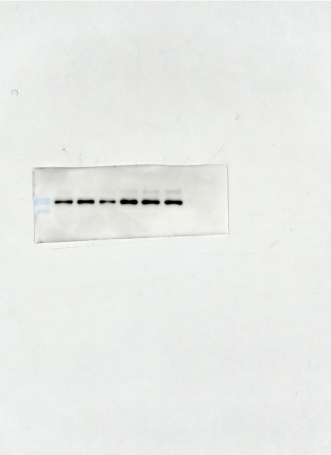

FIG.5 Occludin

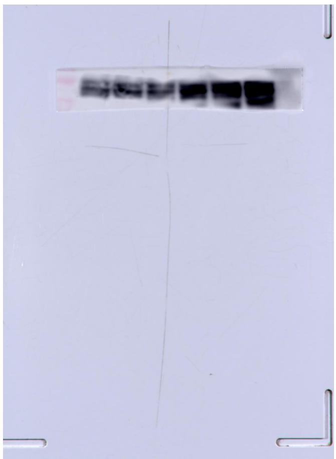

FIG.5 GAPDH

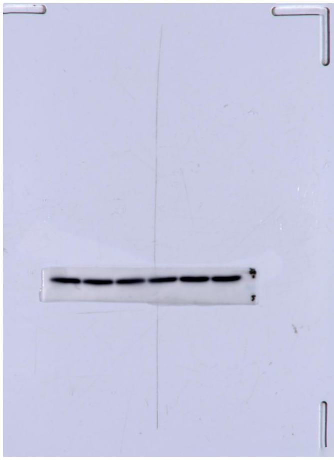

FIG.5 Zo1

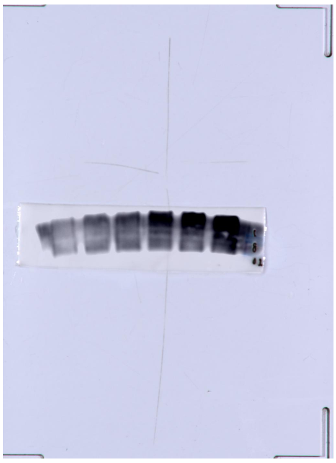

FIG.5 N-cadherin

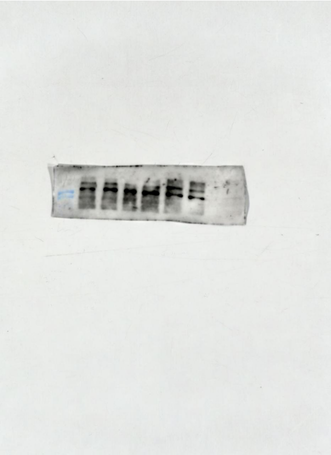

FIG.6 GAPDH(concentration)

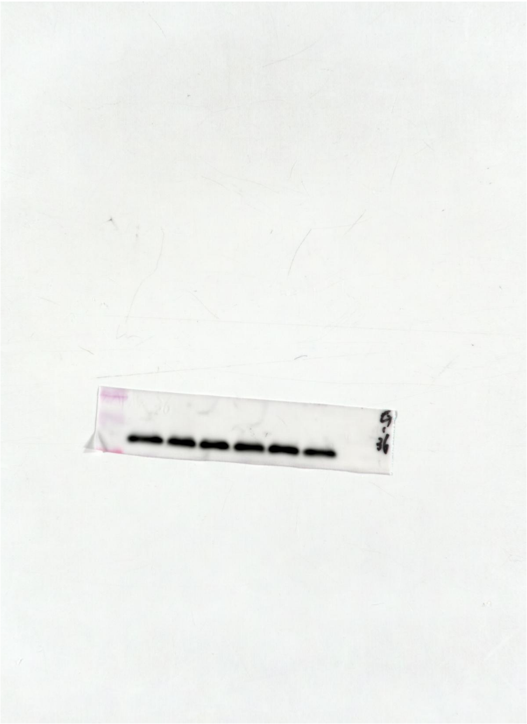

FIG.6 SGK1(concentration)

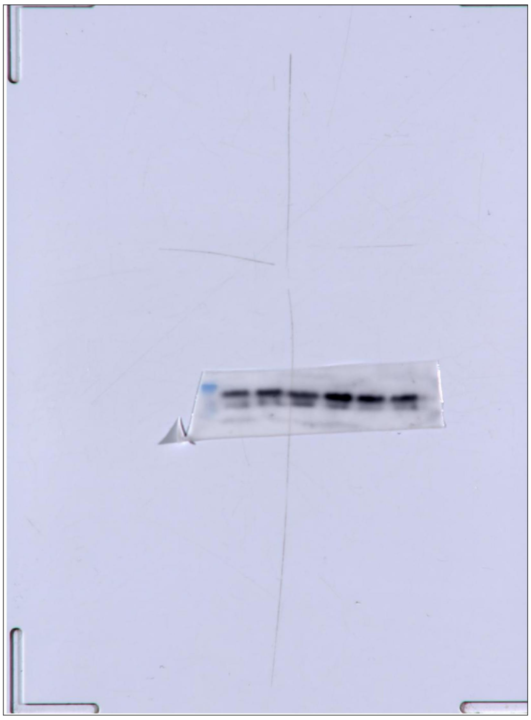

FIG.6 GAPDH(time)

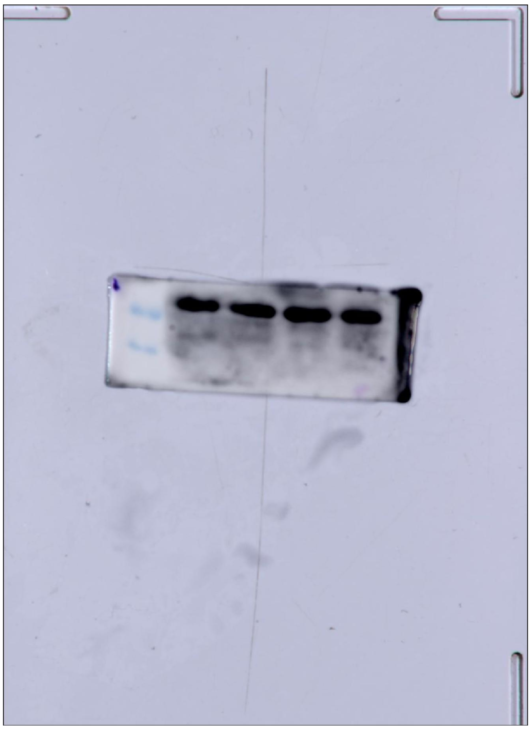

FIG.6 SGK1(time)

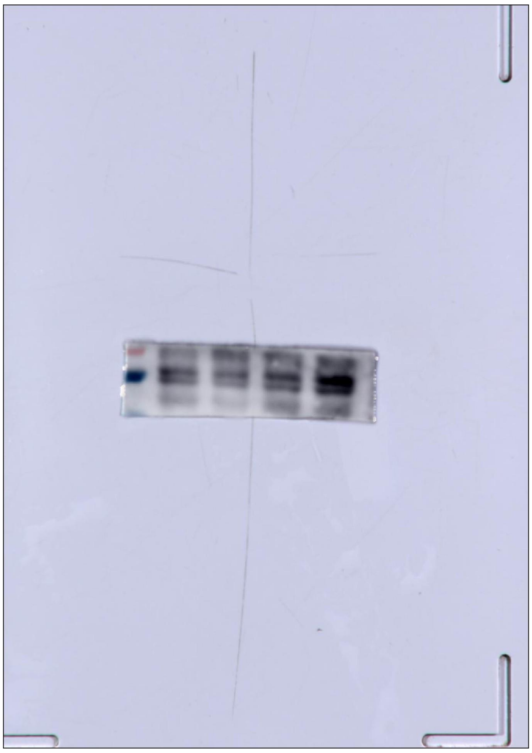

FIG. 7 GAPPDH

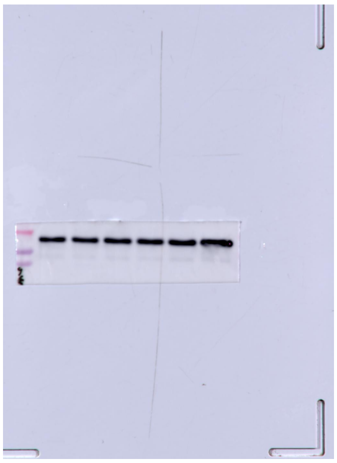

FIG. 7 SGK1

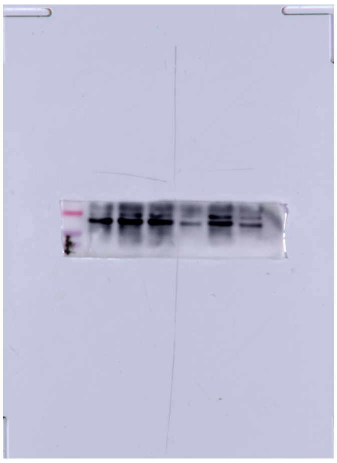

FIG. 7 NC group

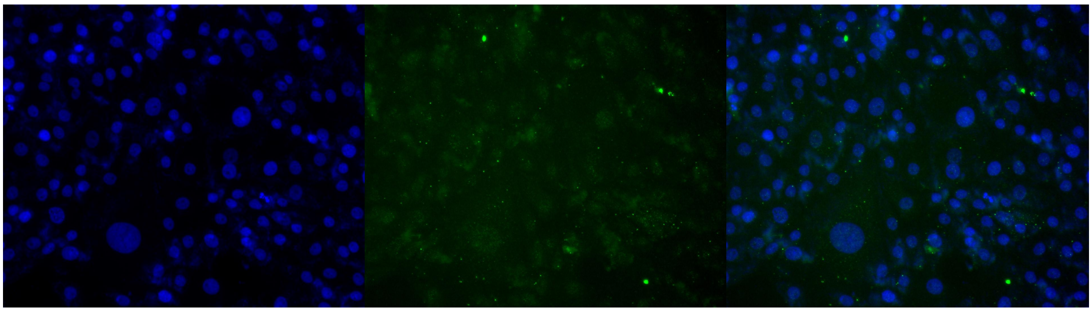

FIG. 7 E_2 group

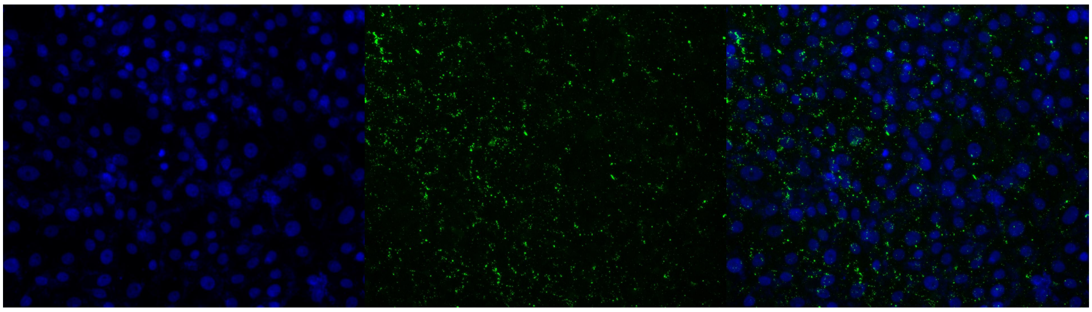

FIG. 7 SI-NC group

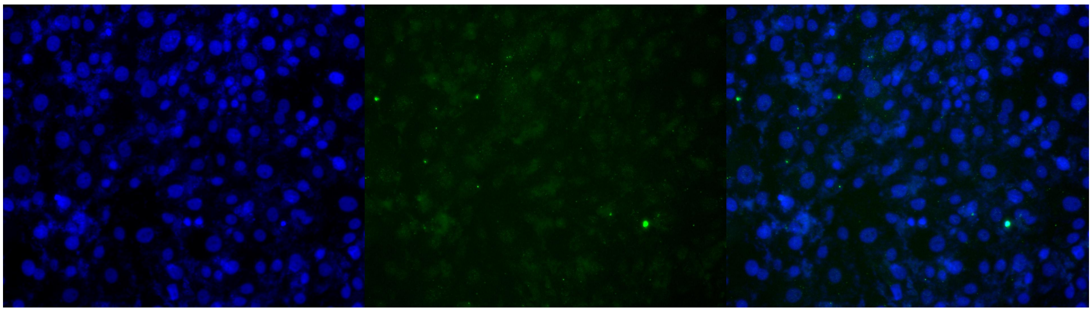

FIG. 7 SI-SGK1 group

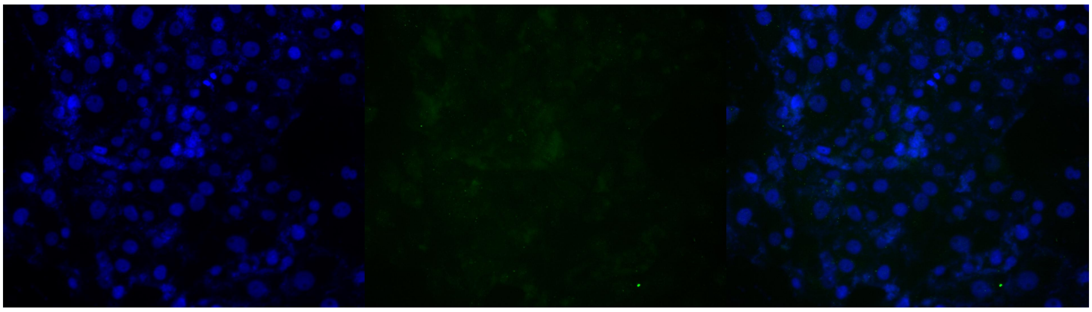

FIG. 7 E_2+SI-NC group

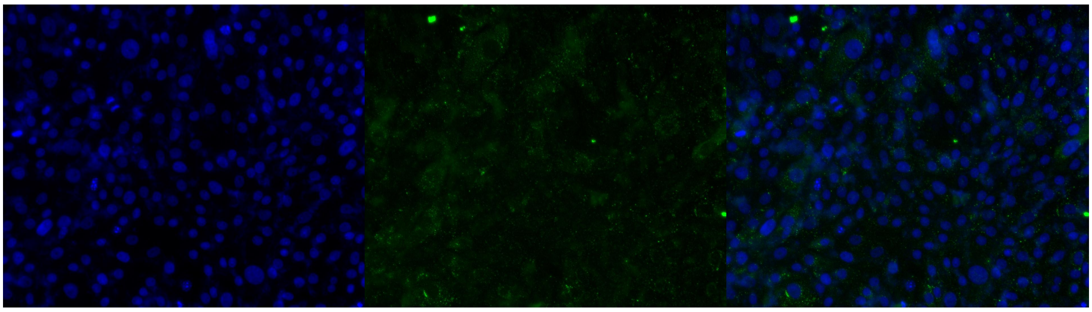

FIG. 7 E_2+SI-SGK1 group

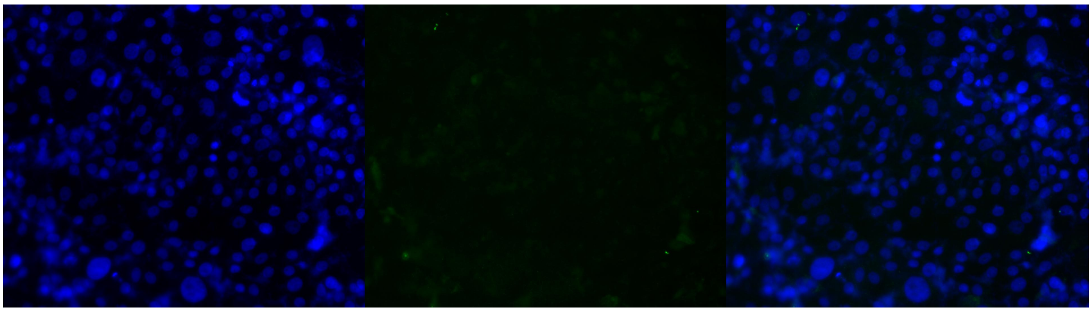

FIG.8 Bax

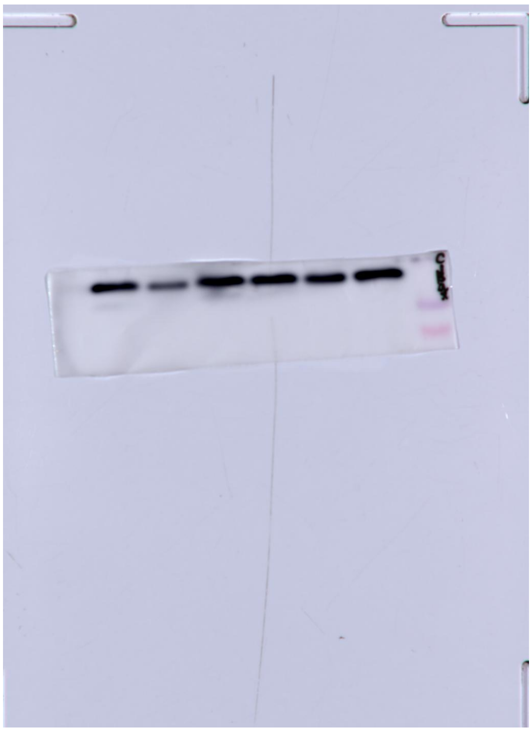

FIG.8 caspase-3

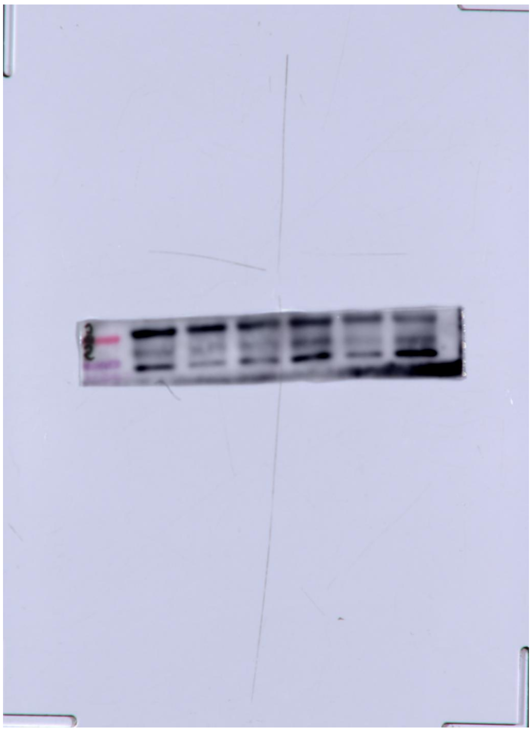

FIG.8 Bcl-2

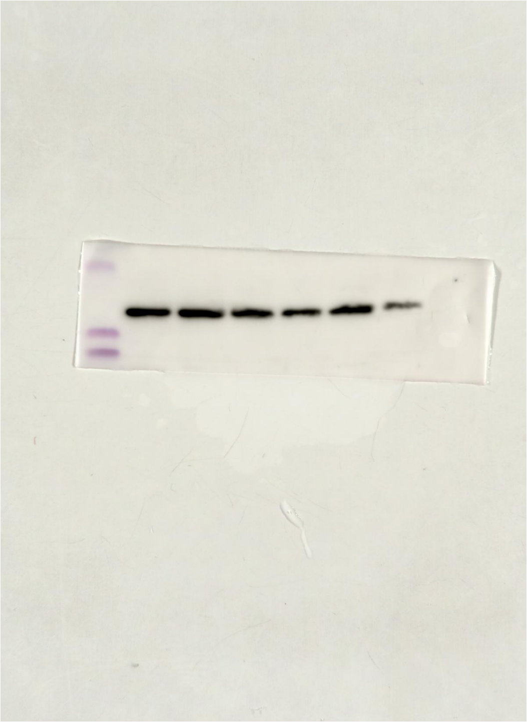

FIG.8 GAPDH

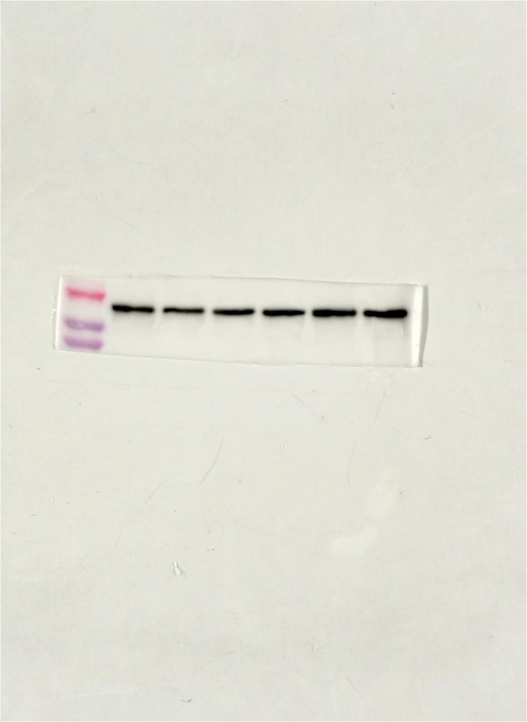

FIG.9 E-CADHERIN

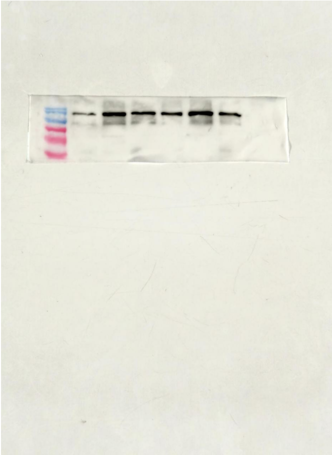

FIG.9 OCCLUDIN

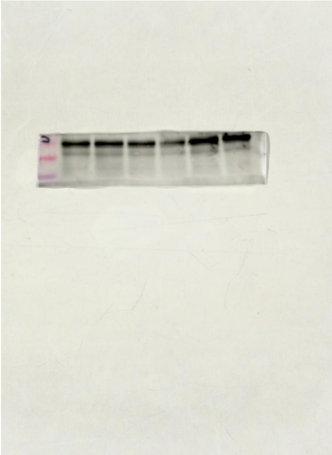

FIG.9 GAPDH

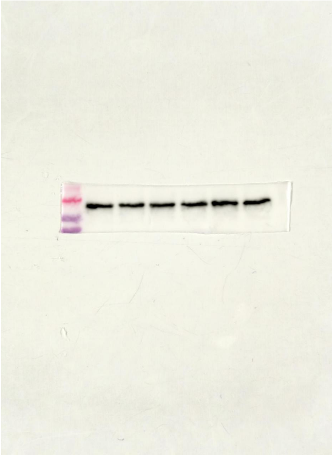

FIG.9 ZO1

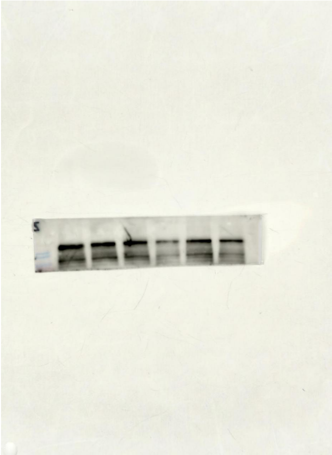

FIG.9 N-CADHERIN

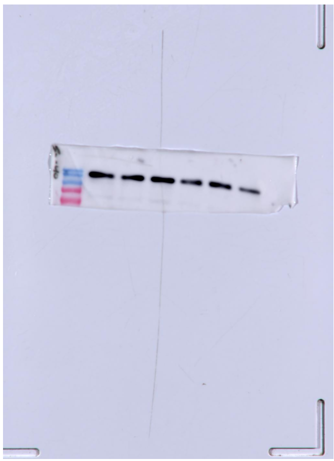

FIG.9 NC-0h

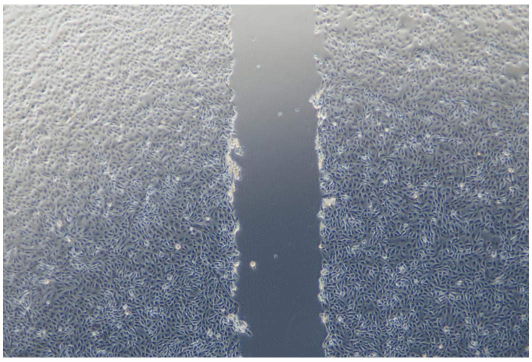

FIG.9 SI-SGK1-0h

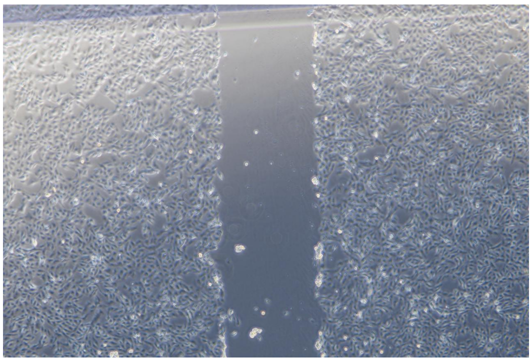

FIG.9 E_2-0h

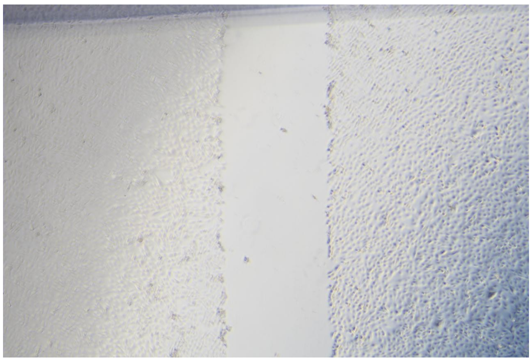

FIG.9 E_2+SI-NC-0h

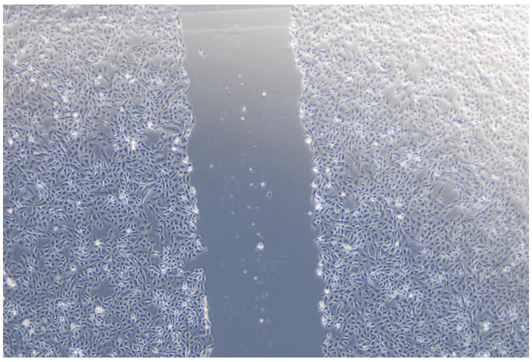

FIG.9 SI-NC-0h

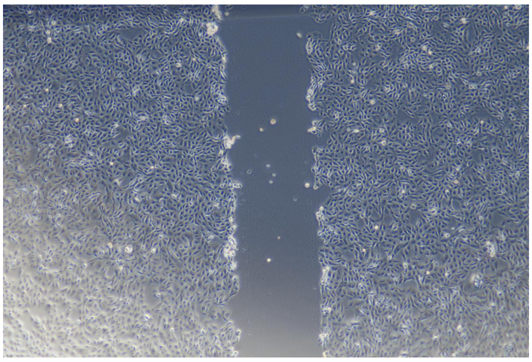

FIG.9 E_2+SI-SGK1-0h

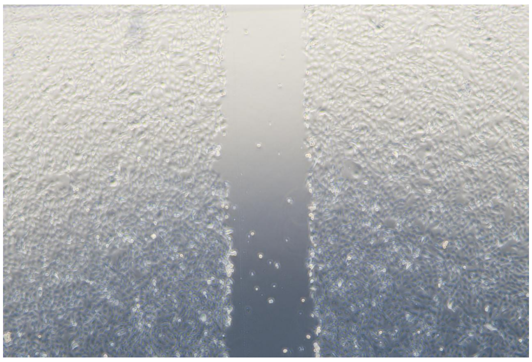

FIG.9 NC-48h

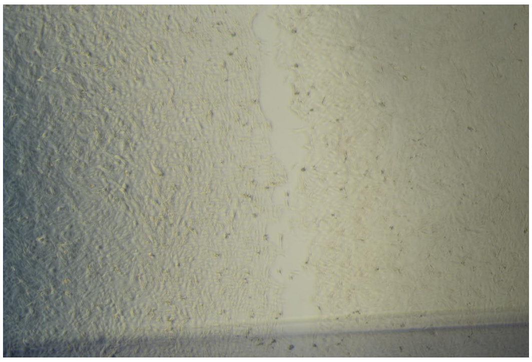

FIG.9 SI-SGK1-48h

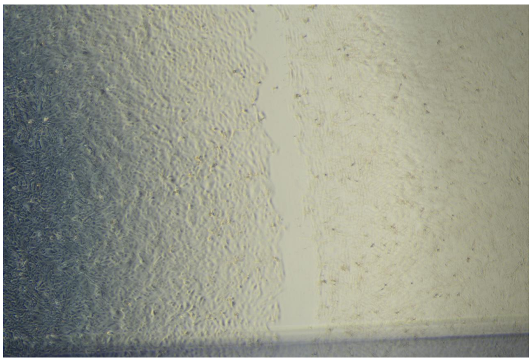

FIG.9 E_2-48h

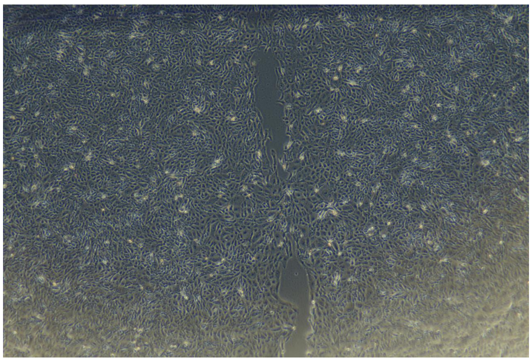

FIG.9 E_2+SI-NC-48h

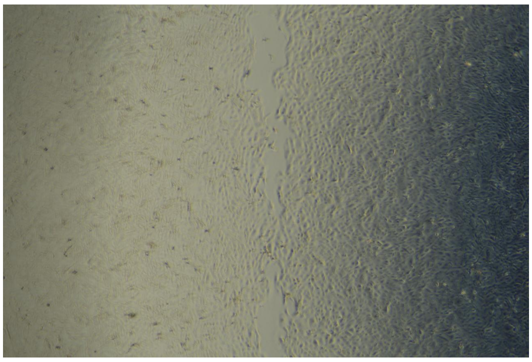

FIG.9 SI-NC-48h

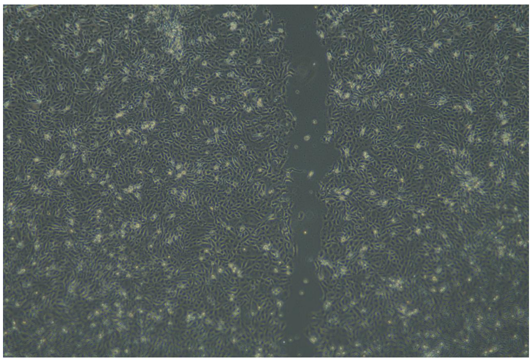

FIG.9 E_2+SI-SGK1-48h

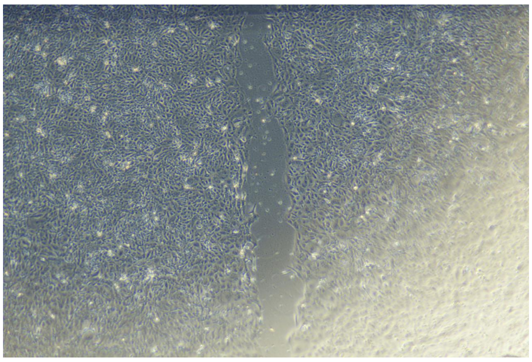

Supplement: Supplementary file 1 [file cells-15-00558-s001.zip › Supplementary figure S1.pdf]
