# Supplementary material for: SGK1 Is Upregulated in Retained Placenta and Mediates Estradiol Effects in Bovine Endometrial Cells
Source: Cells. 2026 Mar 20;15(6):558. doi: 10.3390/cells15060558 (PMC13025382; doi:10.3390/cells15060558)

Supplementary Figure . Negative controls for immunohistochemical staining.

NC（No primary antibody）　　　　RP（No primary antibody）

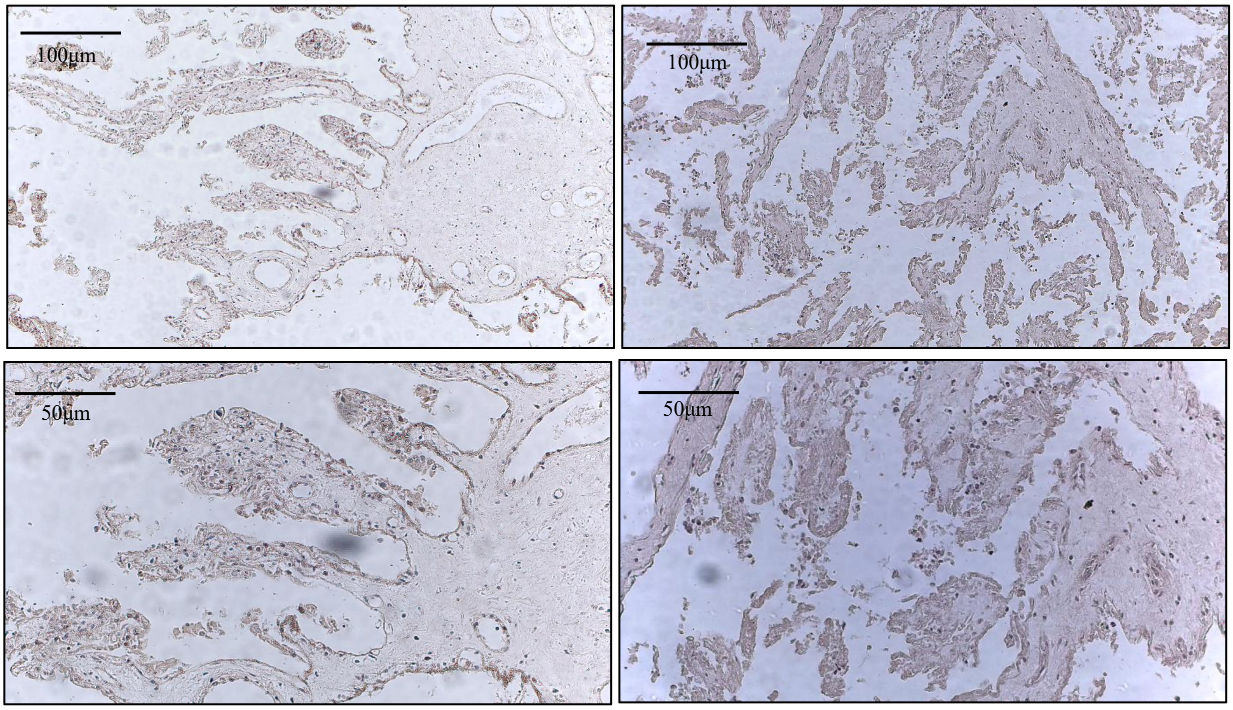

Negative controls for immunohistochemical staining. Representative images of fetal cotyledonary tissues from RP cows stained under identical conditions with omission of the primary antibody (replaced with PBS). No specific brown DAB signal was detected in any of these control sections, while hematoxylin counterstaining clearly visualizes the tissue architecture.

Supplementary Figure . Negative controls for Western blot.staining.

GAPDH

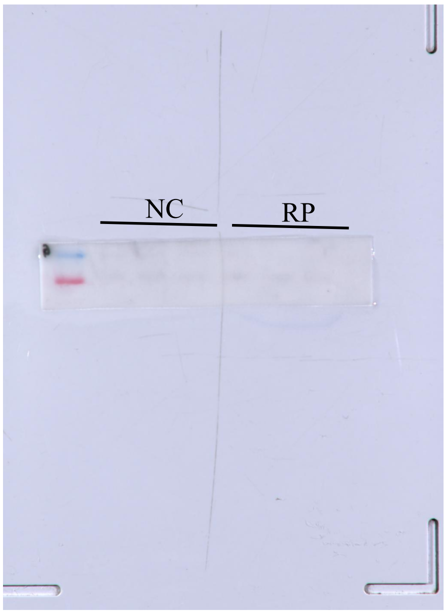

BAX

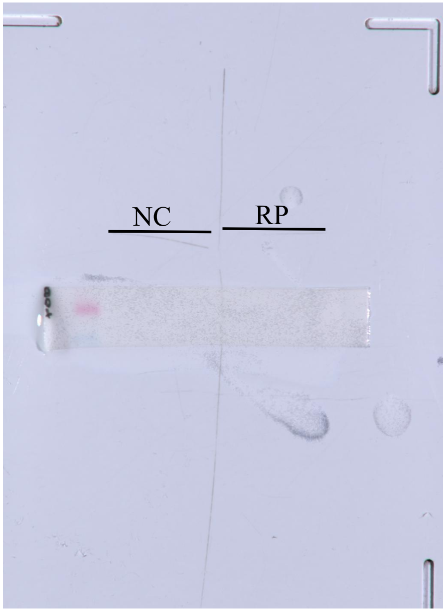

SGK1

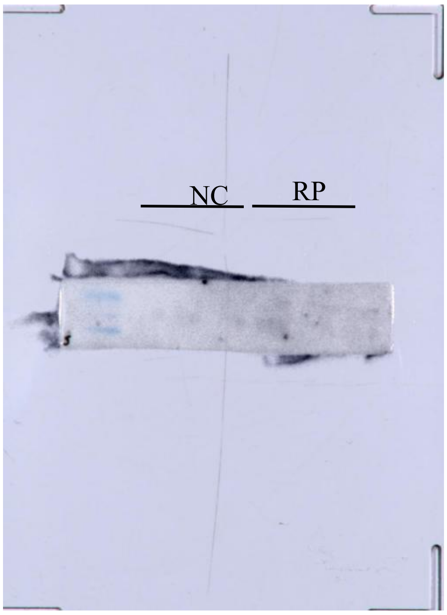

BCL2

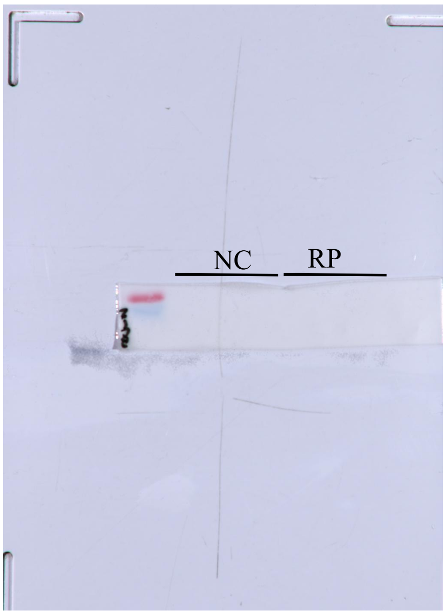

CASEPASE-3

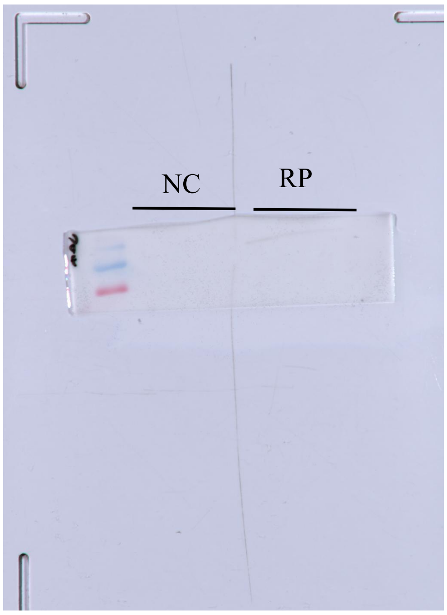

OCCLUDIN

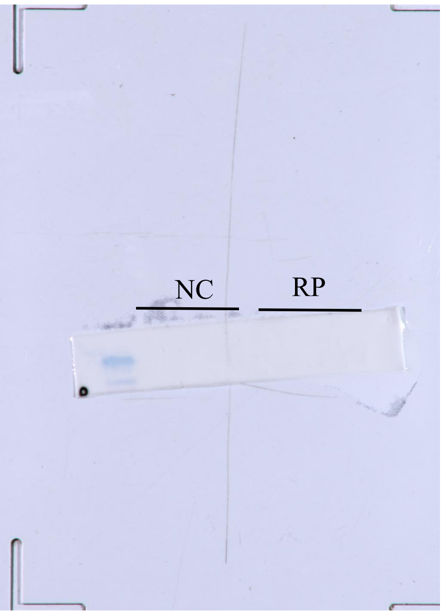

E-CADHERIN

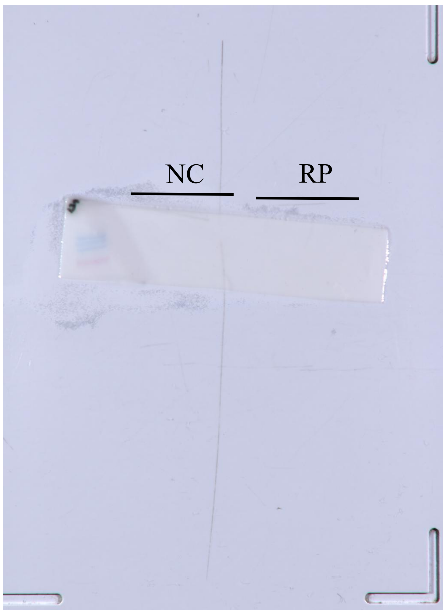

ZO1

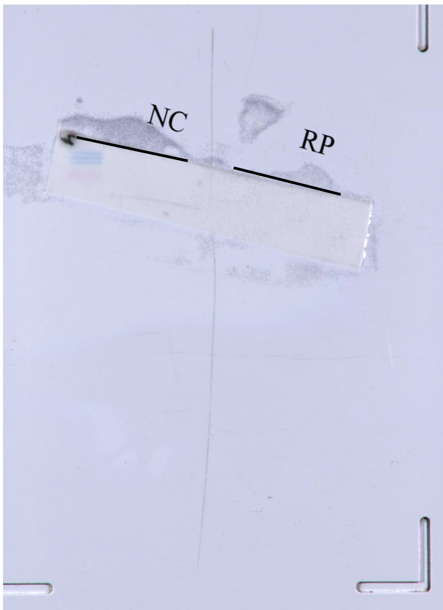

N-CADHERIN

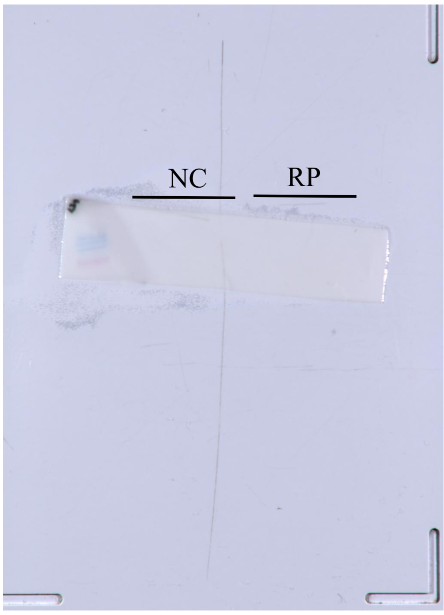

Supplement: Supplementary file 1 [file cells-15-00558-s001.zip › Supplementary figure S2.pdf]
