# Supplementary material for: SGK1 Is Upregulated in Retained Placenta and Mediates Estradiol Effects in Bovine Endometrial Cells
Source: Cells. 2026 Mar 20;15(6):558. doi: 10.3390/cells15060558 (PMC13025382; doi:10.3390/cells15060558)
Supplement: Supplementary file 1 [file cells-15-00558-s001.zip › Supplementary Table S1.pdf]

Table S1 Information of primer sequences for PCR and si-RNA sequences.

Name	Forward Primer (5'-3')	Reverse Primer (5'-3')	Tm (◦C)
SGK1	GGACAAAGAGGCTGGGTG	TTGACGCTGGCTGTGAGA	55.70
BAX	GACAGGGGCCCTTTTGCTTC	CCACAGCTGCGATCATCCTCT	60.85
BCL-2	TCATGTGTGGAGAGCGTC	CTCCACAAAGGCGTCCCAG	56.00
Caspase-3	GTCAGTCCTGGGCTGGTTC	TGCTTCCATGTAAGATCTTTGTCT	54.50
ZO1	GTCCATGACTCCTGACGGTT	GGTTTTAGGATCACCCGACGA	59.15
Occludin	GGTGCGCCCTCCAGATTG	CTGGATGACATGGCTGGTGT	60.05
E-cadherin	GGCTGGACCGTGAGAGTTTT	GCAGTTGTGCTCAAGCCTTC	58.50
N-cadherin	AGCCCGGTTTCATTTGAGGG	TCGTCAGCATCAATCGCAGT	60.00
EFCAB7	GAGTCAAGCTGAAGGAAAACCA	TTCACCAGTCCACCCAAACG	60.00
SYT3	TGACTTTGACCGCTTCTCCC	AGATCTGCCTTTTCCGAGCC	60.09
PCDH7	ATCGTGAAGGGGAGCGTGTA	CGGTCTATACGGTGCAGGAC	59.20
RIC3	GGGGCCTAATGGTGAGAGAG	TCTTCGGGATAACCTTCCCAG	59.00
MYH7	CGCAAGTCAGAGAAGGAG	GTCGGAGATGGAGAAGATG	51.10
KCNAB3	GATGAGACAGCAGAGGATG	TTGGTGGTGATGACATAGC	50.50
MTUS1	GAATCTGCGACTTCCTTCA	GAATCTGCGACTTCCTTCA	53.00
SYT4	TTCCGTTCAGCACCTCAGAC	CCCCGTCCTGAAGATTTCCTA	60.20
PIGR	TAGTGTTCAGCGTTGTCAT	GATTGCCGTCTCTTCATTG	49.90
NELL2	CGATGAGTGCTCTGATGG	AATCTGACCGTTGTGCTTA	52.50
CCL5	CATGGCAGCAGTTGTCTTTATCA	CTCGCACCCACTTCTTCTCT	58.50
LMAN2L	AAATTTGTGGGGCTGGGAGTAT	GGGAACACCCGCTGGACTC	61.00
GPR82	AGCATCACCACCCTGAACAA	GTGGATGGTGCCTTTGTCTGTA	59.00
GPR4	CATTGCTGACCTGCTGTA	GCGATGCTGATGTAGATGT	50.00
CUBN	TACCATTGCGACAGGTGCTT	AGCCTGGGGAGTAGAGGAAG	58.20
SGK1 si-1	CAGCUGAGAUGUACGACAATT	UUGUCGUACAUCUCAGCUGTT	56.10
SGK1 si-2	CCUCCAGUUGAAGCCAAAUAUTT	AUAUUUGGCUUCAACUGGAGGTT	56.60
SGK1 si-3	GGAUGACUUUAUGGAGAUUAATT	UUAAUCUCCAUAAAGUCAUCCTT	53.00
